# Supplementary material for: Experimental observation of classical analogy of topological entanglement entropy
Source: Nat Commun. 2019 Apr 5;10:1557. doi: 10.1038/s41467-019-09584-1 (PMC6450868; doi:10.1038/s41467-019-09584-1)
Supplement: Supplementary file 1 — Supplementary Information [file 41467_2019_9584_MOESM1_ESM.pdf]

# Supplementary Information for

# Experimental observation of classical analogy of topological entanglement entropy

Chen et al.

# Supplementary Figures

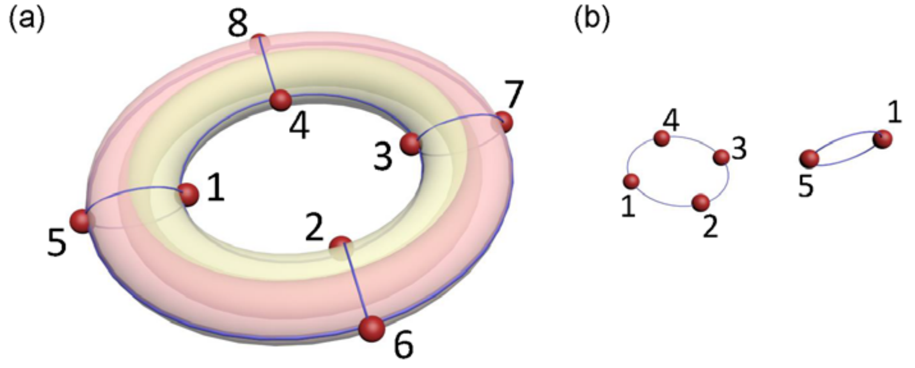


**Supplementary Figure 1.** The loop operators in the 8-spins square lattice. Each red sphere represents one spin. (a) The schematic representation of 8-spins square lattice. (b) Two loops along x and y-directions are presented.

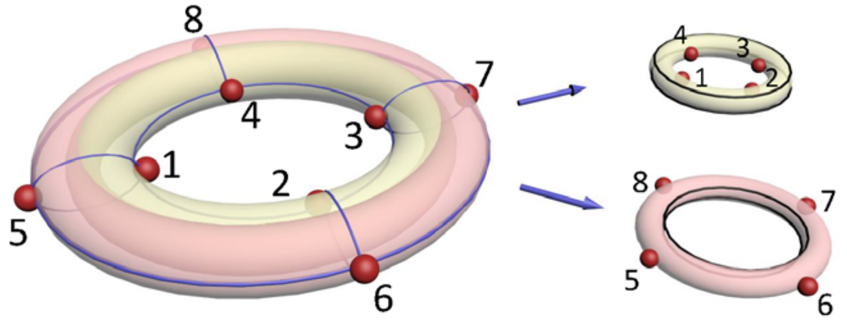


**Supplementary Figure 2.** The separation of 8-spins square lattice. Each red sphere represents one spin. One subsystem (yellow regions) contains the spin 1, 2, 3 and 4, and the remaining (pink region) contains the spin 5, 6, 7 and 8. Two boundaries are required to separate these two subsystems, and are represented by black solid lines.

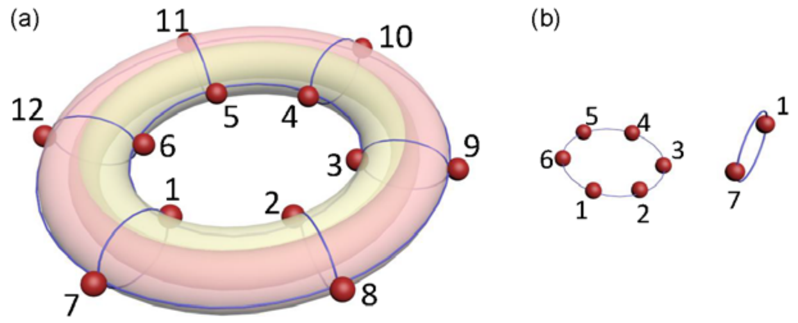


**Supplementary Figure 3.** The loop operators in the 12-spins square lattice. Each red sphere represents one spin. (a) The schematic representation of 12-spins square lattice. (b) Two loops along x and y-directions are presented.

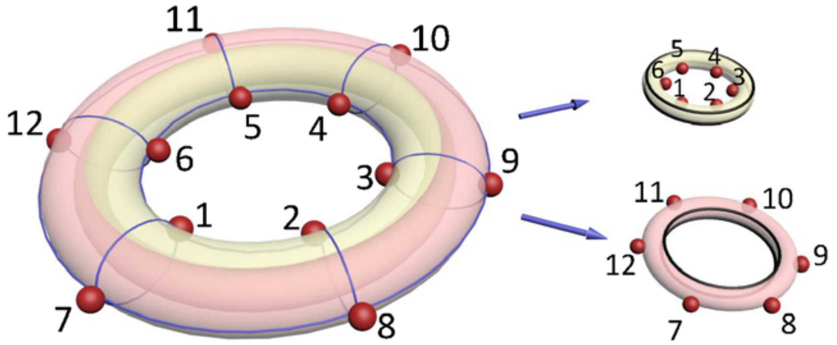


**Supplementary Figure 4.** The separation of 12-spins square lattice. Each red sphere represents one spin. One subsystem (yellow region) contains the spin 1, 2, 3, 4, 5 and 6, and the remaining (pink region) contains the spin 7, 8, 9, 10, 11 and 12. Two boundaries are required to separate these two subsystems, and are represented by black solid lines.

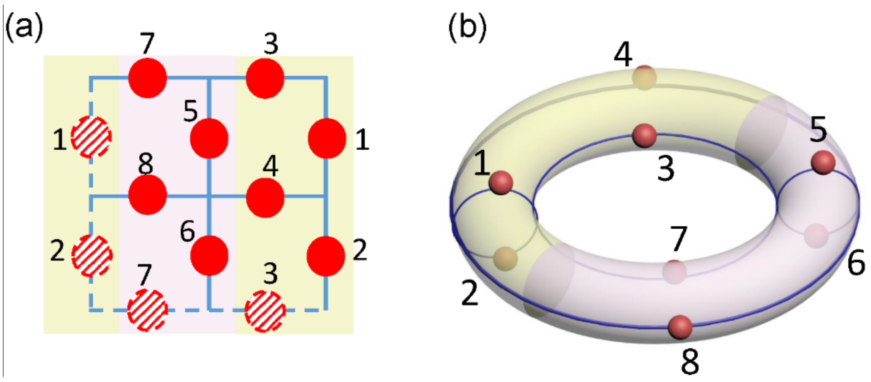


**Supplementary Figure 5.** The schematic representation of toric code model with 8 spins. Each red sphere represents one spin. (a) The unfolded forms of the torus structure. The shadow spheres are drawn to show the periodic boundary. Periodic boundary conditions are chosen in both x and y directions. (b) The geometry of the toric code model. To obtain the entanglement entropy for this model, we separate the total system into two subsystems, one (yellow region) contains the spin 1, 2, 3 and 4, and the other (pink region) contains the spin 5, 6, 7 and 8. Two disconnected boundaries exist between two subsystems (yellow and pink regions), and the lengths for each boundary is 2.

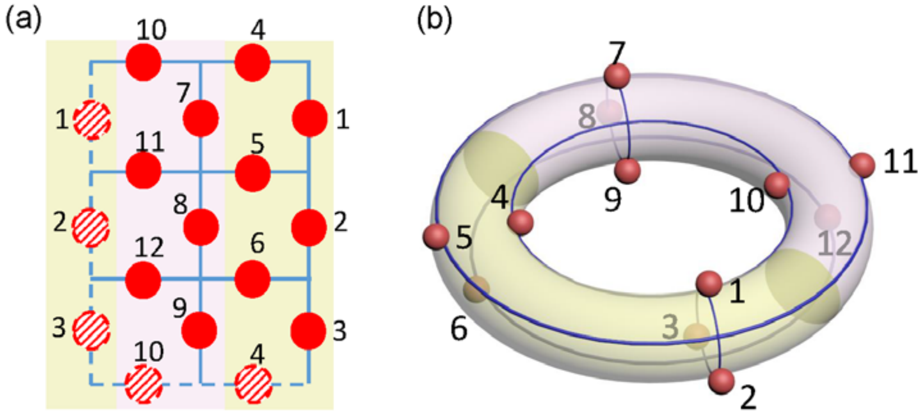


**Supplementary Figure 6.** The schematic representation of toric code model with 12 spins. Each red sphere represents one spin. (a) The unfolded forms of the torus structure. The shadow spheres are drawn to show the periodic boundary. Periodic boundary conditions are chosen in both x and y directions. (b) The geometry of the toric code model. To obtain the entanglement entropy for this model, we separate the total system into two subsystems, one (yellow region) contains the spin 1, 2, 3, 4, 5 and 6, and the other (pink region) contains the spin 7, 8, 9, 10, 11 and 12. Two disconnected boundaries exist between two subsystems (yellow and pink regions), and the length for each boundary is 3.

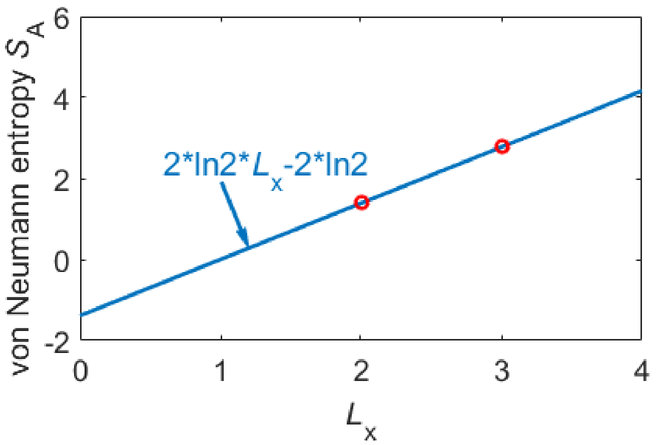


**Supplementary Figure 7.** The von Neumann entropies for MESs of 8 and 12-spins toric code model. Red circles represent the entropies values for the corresponding 8 and 12-spins systems. The numerical fitting curve is addressed as a blue solid line.

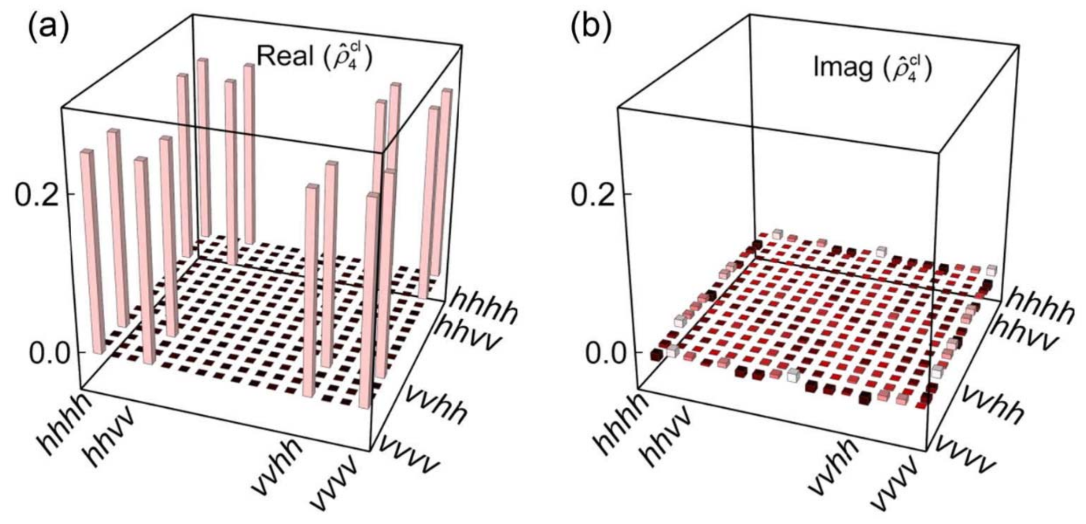


**Supplementary Figure 8.** Tomographic reconstruction of the experimental analogy of the density matrix for $\hat{\rho}_4^{cl}$. (a) Experimental real part of the analogy density matrix. (b) Experimental imaginary part. Source data are provided as a Source Data file.

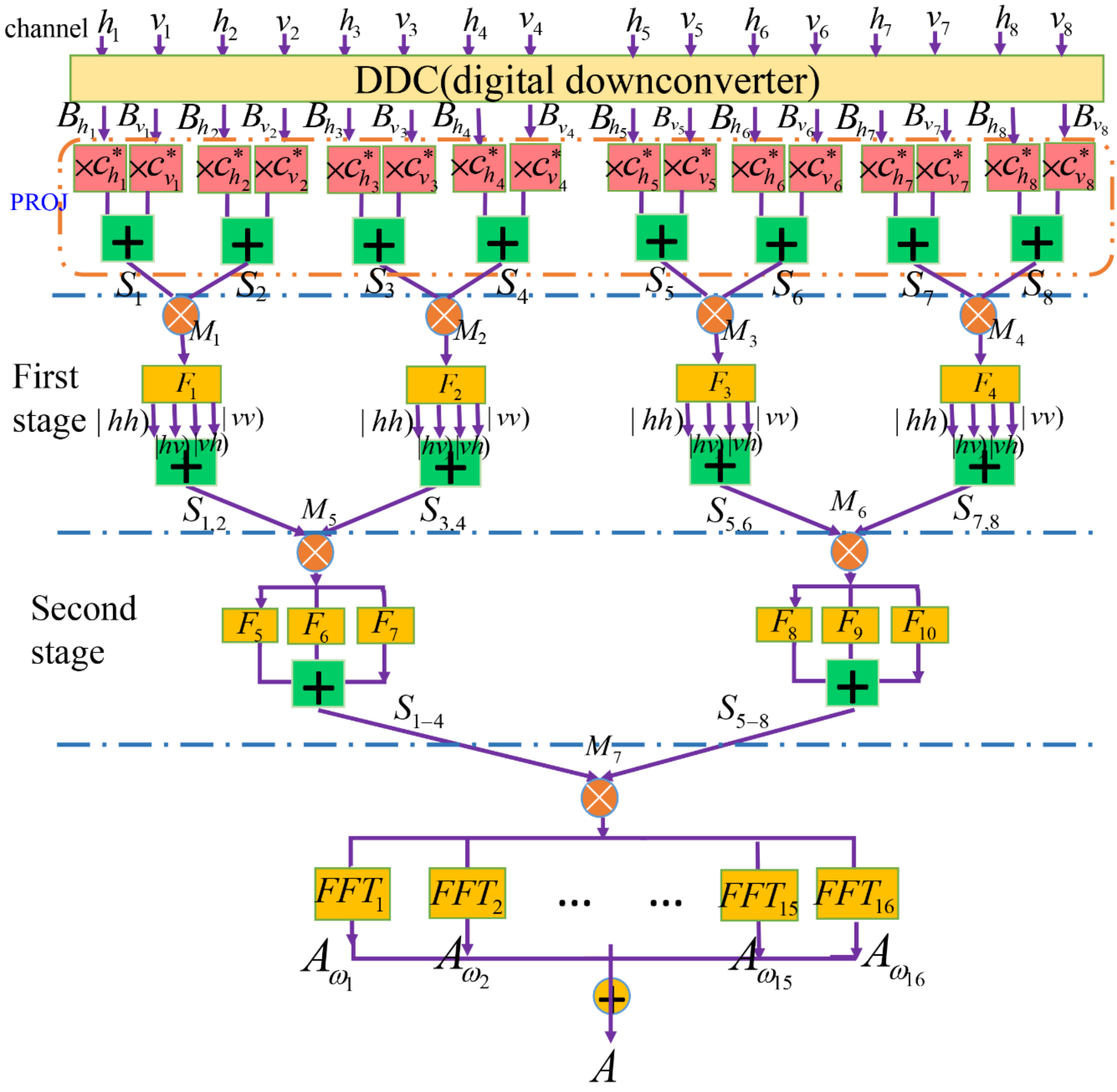


**Supplementary Figure 9.** The designed circuit to construct $|\psi_8^{\text{cl}}\rangle$ in the digital signal processing (DSP) module. All 16 signals in the channels $\{h_1, v_1, \cdots, h_8, v_8\}$ fed into the DSP are digitally down-converted to their own frequencies set in Supplementary Equation 21. After a measuring process through the PROJ part followed by the designated stage-by-stage mixing and filtering processes for multiplex signals, the 16 desired frequency components $A_{\omega_1}$, $A_{\omega_2}$,…, $A_{\omega_{16}}$ are selected by a collection of FFT-based digital filters $\{FFT_1, \ldots, FFT_{16}\}$, respectively. Each filtered frequency component is identified with a superposition term of the desired state, and the sum of complex amplitudes for all filtered frequency components is denoted as *A*.

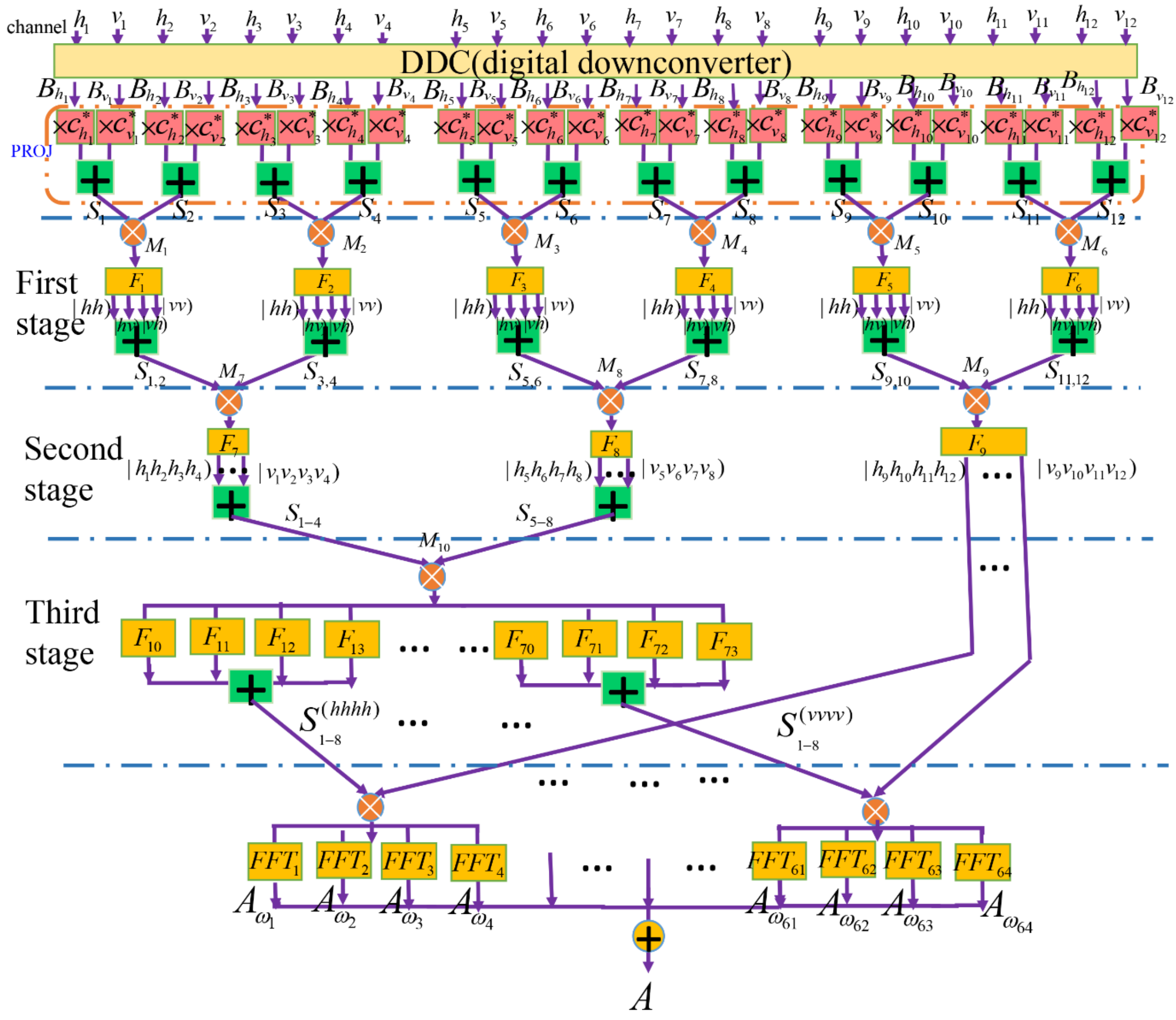


**Supplementary Figure 10.** The designed circuit to construct $|\psi_{12}^{\mathrm{cl}}\rangle$ in the digital signal processing (DSP) module. All 24 signals in the channels $\{h_1, v_1, \cdots, h_{12}, v_{12}\}$ fed into the DSP are digitally down-converted to their own frequencies set in Supplementary Equation 23. After a measuring process through the PROJ part followed by the designated stage-by-stage mixing and filtering processes for multiplex signals, the 64 desired frequency components $A_{\omega_1}, A_{\omega_2}, \ldots, A_{\omega_{64}}$ are selected by a collection of FFT-based digital filters $\{FFT_1, \ldots, FFT_{64}\}$, respectively. Each filtered frequency component is identified with a superposition term of the desired state, and the sum of complex amplitudes for all filtered frequency components is denoted as *A*.

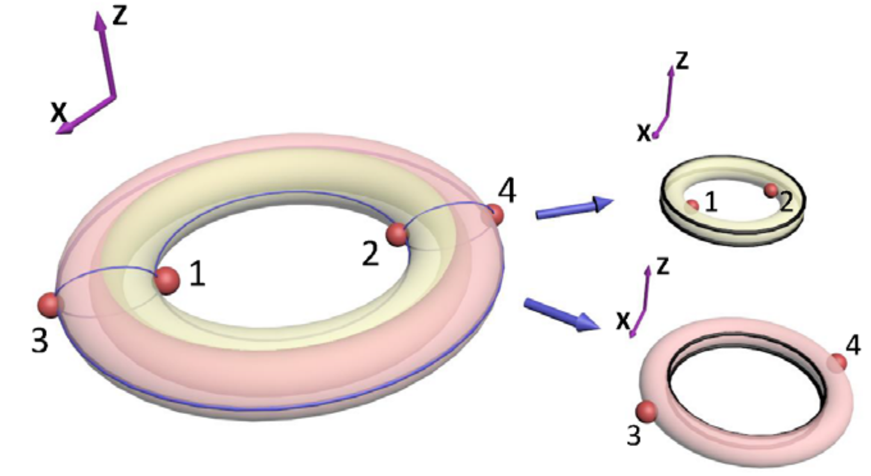


**Supplementary Figure 11.** The separation of 4-spins square lattice. Each red sphere represents one spin. One subsystem (yellow region) contains the spin 1 and 2, and the remaining (pink region) contains the spin 3 and 4. Two boundaries are required to separate these two subsystems, and are represented by black solid lines. The external fields along x and z directions which are presented by purple arrows.

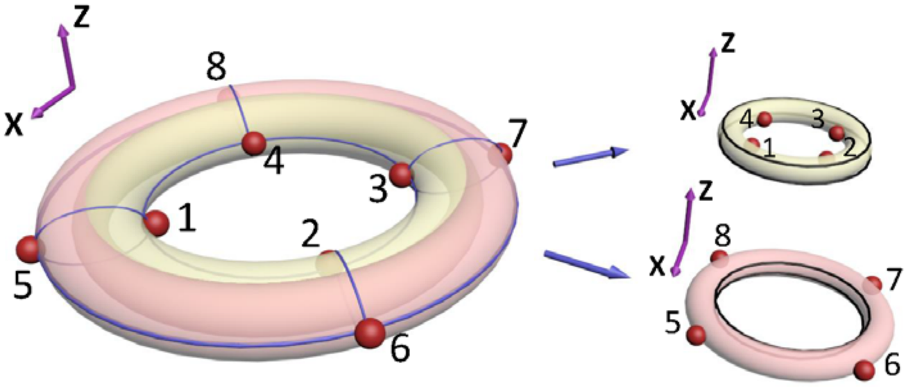


**Supplementary Figure 12.** The separation of 8-spins square lattice. Each red sphere represents one spin. One subsystem (yellow region) contains the spin 1, 2, 3 and 4, and the remaining (pink region) contains the spin 5, 6, 7 and 8. Two boundaries are required to separate these two subsystems, and are represented by black solid lines. The external fields along x and z directions which are presented by purple arrows.

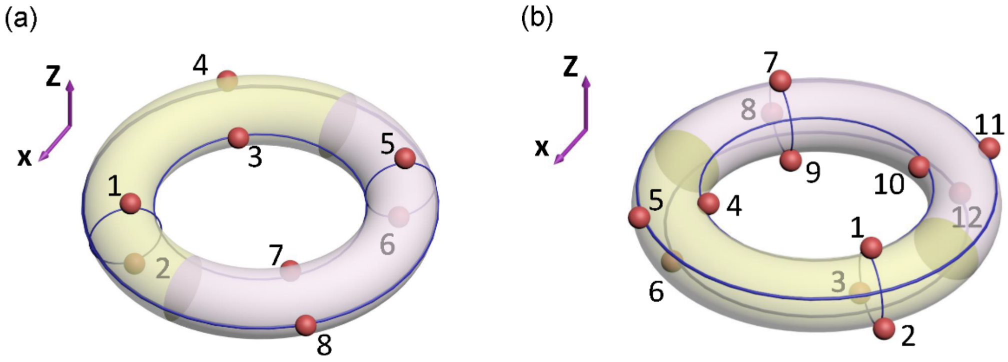


**Supplementary Figure 13.** The separation of toric code model with external fields. Each red sphere represents one spin. (a) One subsystem (yellow region) contains the spin 1, 2, 3 and 4, and the remaining (pink region) contains the spin 5, 6, 7 and 8. (b) One subsystem (yellow region) contains the spin 1, 2, 3, 4, 5 and 6, and the remaining (pink region) contains the spin 7, 8, 9, 10, 11 and 12. Two boundaries are required to separate these two subsystems. The external fields along x and z directions which are presented by purple arrows.

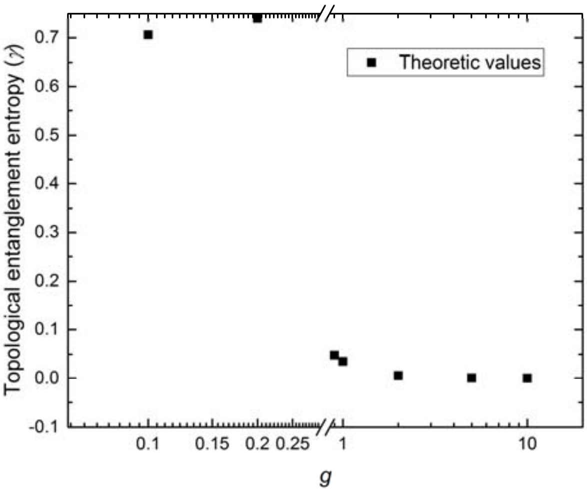


**Supplementary Figure 14.** The topological entanglement entropy for the toric code model with external fields. When the strength $g < 0.34$, the system belongs to the $Z_2$ topological phase, with $\gamma = \ln 2$; when the strength $g > 0.34$, the system belongs to the topologically trivial phase, with $\gamma = 0$.

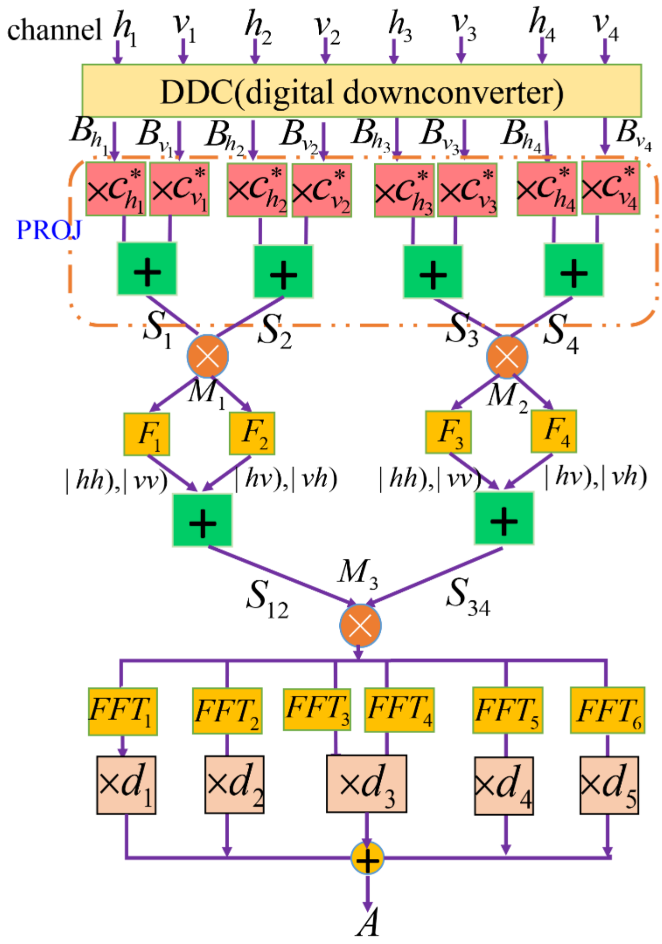


**Supplementary Figure 15.** The designed work flow for the analogy of $|\Xi\rangle_4$ with small $g$ in the DSP module. All 8 signals in the channels $\{h_1, v_1, \cdots, h_4, v_4\}$ fed into the DSP are digitally down-converted to their own frequencies set in Supplementary Equation 45. After a measuring process through the PROJ part followed by several mixing and filtering processes for multiplex signals, the desired frequency components selected by 6 FFT-based digital filters $\{FFT_1, \cdots, FFT_6\}$ are adjusted by 5 modulators $\{d_1, \ldots, d_5\}$ respectively, and their joint complex amplitude is denoted as *A*.

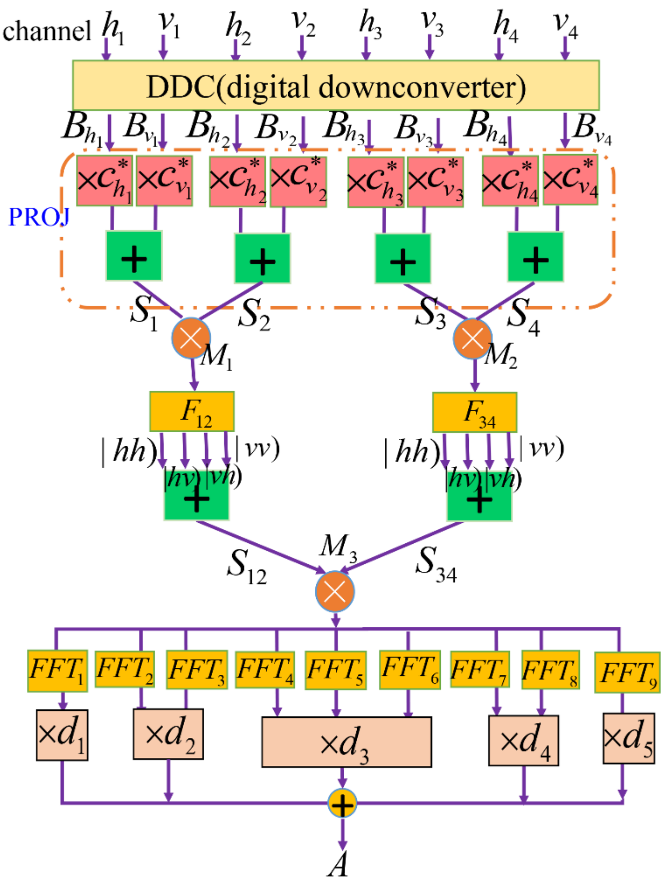


**Supplementary Figure 16.** The designed work flow for analogy of state $\left|\Phi_g\right\rangle_4$ with large $g$ in the DSP module. All 8 signals in the channels $\{h_1, v_1, \cdots, h_4, v_4\}$ fed into the DSP are digitally down-converted to their own frequencies set in Supplementary Equation 45. After a measuring process through the PROJ part followed by several mixing and filtering processes for multiplex signals, the desired frequency components selected by 9 FFT-based digital filters $\{FFT_1, \cdots, FFT_9\}$ are adjusted by 5 modulators $\{d_1, \ldots, d_5\}$ respectively, and their joint complex amplitude is denoted as $A$.

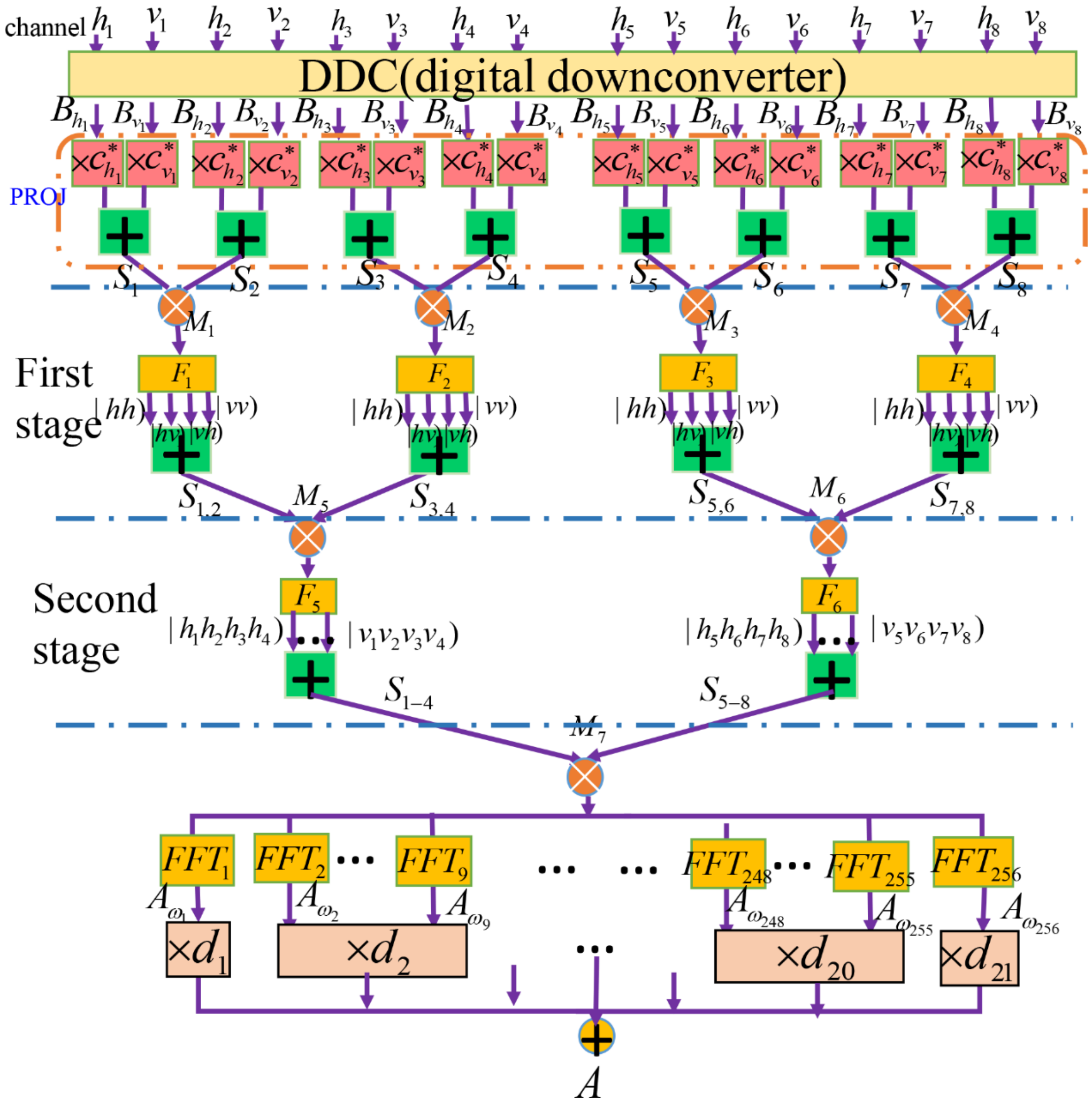


**Supplementary Figure 17.** The designed circuit for analogy of 8-cebit state $\left|\Phi_g\right\rangle_8$ with large $g$ in the DSP module. All 16 signals in the channels $\{h_1, v_1, \cdots, h_8, v_8\}$ fed into the DSP are digitally down-converted to their own frequencies set in Supplementary Equation 21. After a measuring process through the PROJ part followed by the designated stage-by-stage mixing and filtering processes for multiplex signals, the desired frequency components selected by 256 FFT-based digital filters denoted by $\{A_{\omega_1}, A_{\omega_2}, \ldots, A_{\omega_{256}}\}$ are adjusted by 21 modulators $\{d_1, \ldots, d_{21}\}$ respectively. Each filtered frequency component is identified with a superposition term of the desired state, and the sum of complex amplitudes for all filtered frequency components is denoted as *A*.

# Supplementary Tables

**Supplementary Table 1.** Distinct frequency components (MHz) selected by bandpass filters $F_1$ and $F_2$ in the first stage, and then $F_5$, $F_6$ and $F_7$ in the second stage in Supplementary Figure 9 and their corresponding terms.

| filter | $F_1$ (passband: 3.00—8.00MHz) | | | | $F_2$ (passband: 3.50—10.00MHz) | | | |
|---|---|---|---|---|---|---|---|---|
| selected frequency | 3.25 | 5.60 | 5.52 | 7.87 | 3.93 | 6.92 | 6.67 | 9.66 |
| term | $\|h_1h_2\rangle$ | $\|h_1v_2\rangle$ | $\|v_1h_2\rangle$ | $\|v_1v_2\rangle$ | $\|h_3h_4\rangle$ | $\|h_3v_4\rangle$ | $\|v_3h_4\rangle$ | $\|v_3v_4\rangle$ |
| filter | $F_5$ | $F_6$ (passband: 11.0—13.5MHz) | | | | | | $F_7$ |
| selected frequency | 7.18 | 12.91 | 12.52 | 12.27 | 12.44 | 12.19 | 11.80 | 17.53 |
| term | $\|h_1h_2h_3h_4\rangle$ | $\|h_1h_2v_3v_4\rangle$ | $\|h_1v_2h_3v_4\rangle$ | $\|h_1v_2v_3h_4\rangle$ | $\|v_1h_2h_3v_4\rangle$ | $\|v_1h_2v_3h_4\rangle$ | $\|v_1v_2h_3h_4\rangle$ | $\|v_1v_2v_3v_4\rangle$ |

**Supplementary Table 2.** Distinct frequency components (MHz) selected by bandpass filters $F_3$ and $F_4$ in the first stage, and then $F_8$, $F_9$ and $F_{10}$ in the second stage in Supplementary Figure 9 and their corresponding terms.

| filter | $F_3$ (passband: 4.20—11.50MHz) | | | | $F_4$ (passband: 5.00—13.00MHz) | | | |
|---|---|---|---|---|---|---|---|---|
| selected frequency | 4.75 | 7.86 | 7.85 | 10.96 | 5.89 | 8.84 | 9.14 | 12.09 |
| term | $\|h_5h_6\rangle$ | $\|h_5v_6\rangle$ | $\|v_5h_6\rangle$ | $\|v_5v_6\rangle$ | $\|h_7h_8\rangle$ | $\|h_7v_8\rangle$ | $\|v_7h_8\rangle$ | $\|v_7v_8\rangle$ |
| filter | $F_8$ | $F_9$ (passband: 16.00—18.00MHz) | | | | | | $F_{10}$ |
| selected frequency | 10.64 | 16.84 | 16.70 | 17.00 | 16.69 | 16.99 | 16.85 | 23.05 |
| term | $\|h_5h_6h_7h_8\rangle$ | $\|h_5h_6v_7v_8\rangle$ | $\|h_5v_6h_7v_8\rangle$ | $\|h_5v_6v_7h_8\rangle$ | $\|v_5h_6h_7v_8\rangle$ | $\|v_5h_6v_7h_8\rangle$ | $\|v_5v_6h_7h_8\rangle$ | $\|v_5v_6v_7v_8\rangle$ |

**Supplementary Table 3.** Frequencies of the components { $A_{\omega_1}, A_{\omega_2} \ldots, A_{\omega_{16}}$ } selected by the FFT digital filter at the bottom of Supplementary Figure 9 and their corresponding terms in Supplementary Equation 21.

| filtered frequency(MHz) | corresponding term | filtered frequency(MHz) | corresponding term |
|---|---|---|---|
| 17.82 | $\|hhhhhhhh\rangle$ | 30.23 | $\|hhhhvvvv\rangle$ |
| 29.75 | $\|hhvvhhvv\rangle$ | 29.76 | $\|hhvvvvhh\rangle$ |
| 29.22 | $\|hvhvhvhv\rangle$ | 29.51 | $\|hvhvvhvh\rangle$ |
| 29.27 | $\|hvvhhvvh\rangle$ | 28.96 | $\|hvvhvhhv\rangle$ |
| 29.13 | $\|vhhvvhhv\rangle$ | 29.44 | $\|vhhvhvvh\rangle$ |
| 29.18 | $\|vhvhvhvh\rangle$ | 28.89 | $\|vhvhhvhv\rangle$ |
| 28.65 | $\|vvhhvvhh\rangle$ | 28.64 | $\|vvhhhhvv\rangle$ |
| 40.58 | $\|vvvvvvvv\rangle$ | 28.17 | $\|vvvvhhhh\rangle$ |

**Supplementary Table 4.** The parameters of the filters $\{F_1, F_2, \ldots, F_6\}$ in the first stage and $\{F_7, F_8, F_9\}$ in the second stage in Supplementary Figure 10.

| filters | filtered frequencies (MHz) |
|---|---|
| $F_1$ | 3.20—12.60 |
| $F_2$ | 4.60—14.00 |
| $F_3$ | 5.70—15.50 |
| $F_4$ | 7.40—16.60 |
| $F_5$ | 9.40—18.00 |
| $F_6$ | 11.00—19.50 |
| $F_7$ | 8.00—27.00 |
| $F_8$ | 13.00—32.00 |
| $F_9$ | 20.78, 24.78, 24.69,… ,36.85 |

**Supplementary Table 5.** Frequencies of the components { $A_{\omega_1}$, $A_{\omega_2}$ …, $A_{\omega_{64}}$ } selected by the FFT digital filter at the bottom of Supplementary Figure 10 and their corresponding terms in Supplementary Equation 22.

| filtered frequency(MHz) | corresponding term | filtered frequency(MHz) | corresponding term |
|---|---|---|---|
| 42.53 | $\|hhhhhhhhhhhh\rangle$ | 67.46 | $\|hhhhhhvvvvvv\rangle$ |
| 59.50 | $\|hhhhvvhhhhvv\rangle$ | 68.61 | $\|hhhhvvvvvvhh\rangle$ |
| 59.49 | $\|hhhvhvhhhvhv\rangle$ | 68.32 | $\|hhhvhvvvvhvh\rangle$ |
| 59.46 | $\|hhhvvhhhhvvh\rangle$ | 68.47 | $\|hhhvvhvvvhhv\rangle$ |
| 59.66 | $\|hhvhhvhhvhhv\rangle$ | 68.37 | $\|hhvhhvvvhvvh\rangle$ |
| 59.63 | $\|hhvhvhhhvhvh\rangle$ | 68.52 | $\|hhvhvhvvhvhv\rangle$ |
| 59.62 | $\|hhvvhhhhvvhh\rangle$ | 68.23 | $\|hhvvhhvvhhvv\rangle$ |
| 76.59 | $\|hhvvvvhhvvvv\rangle$ | 69.38 | $\|hhvvvvvvhhhh\rangle$ |
| 59.98 | $\|hvhhhvhvhhhv\rangle$ | 68.21 | $\|hvhhhvvhvvvh\rangle$ |
| 59.95 | $\|hvhhvhhvhhvh\rangle$ | 68.36 | $\|hvhhvhvhvvhv\rangle$ |
| 59.94 | $\|hvhvhhhvhvhh\rangle$ | 68.07 | $\|hvhvhhvhvhvv\rangle$ |
| 76.91 | $\|hvhvvvhvhvvv\rangle$ | 69.22 | $\|hvhvvvvhvhhh\rangle$ |
| 60.11 | $\|hvvhhhhvvhhh\rangle$ | 68.12 | $\|hvvhhhvhhvvv\rangle$ |
| 77.08 | $\|hvvhvvhvvhvv\rangle$ | 69.27 | $\|hvvhvvvhhvhh\rangle$ |
| 77.07 | $\|hvvvhvhvvvhv\rangle$ | 68.98 | $\|hvvvhvvhhhvh\rangle$ |
| 77.04 | $\|hvvvvhhvvvvh\rangle$ | 69.13 | $\|hvvvvhvhhhhv\rangle$ |
| 60.08 | $\|vhhhhvvhhhhv\rangle$ | 67.99 | $\|vhhhhvhvvvvh\rangle$ |
| 60.05 | $\|vhhhvhvhhhvh\rangle$ | 68.14 | $\|vhhhvhhvvvhv\rangle$ |
| 60.04 | $\|vhhvhhvhhvhh\rangle$ | 67.85 | $\|vhhvhhhvvhvv\rangle$ |
| 77.01 | $\|vhhvvvvhhvvv\rangle$ | 69.00 | $\|vhhvvvhvvhhh\rangle$ |
| 60.21 | $\|vhvhhhvhvhhh\rangle$ | 67.90 | $\|vhvhhhhvhvvv\rangle$ |
| 77.18 | $\|vhvhvvvhvhvv\rangle$ | 69.05 | $\|vhvhvvhvhvhh\rangle$ |
| 77.17 | $\|vhvvhvvhvvhv\rangle$ | 68.76 | $\|vhvvhvhvhhvh\rangle$ |
| 77.14 | $\|vhvvvhvhvvvh\rangle$ | 68.91 | $\|vhvvvhhvhhhv\rangle$ |
| 60.53 | $\|vvhhhhvvhhhh\rangle$ | 67.74 | $\|vvhhhhhhvvvv\rangle$ |
| 77.50 | $\|vvhhvvvvhhvv\rangle$ | 68.89 | $\|vvhhvvhhvvhh\rangle$ |
| 77.49 | $\|vvhvhvvvhvhv\rangle$ | 68.60 | $\|vvhvhvhhvhvh\rangle$ |
| 77.46 | $\|vvhvvhvvhvvh\rangle$ | 68.75 | $\|vvhvvhhhvhhv\rangle$ |

| | | | |
|---|---|---|---|
| 77.66 | $\|vvvhhvvvvhhv\rangle$ | 68.65 | $\|vvvhhvhhhvvh\rangle$ |
| 77.63 | $\|vvvhvhvvvhvh\rangle$ | 68.80 | $\|vvvhvhhhhvhv\rangle$ |
| 77.62 | $\|vvvvhhvvvvhh\rangle$ | 68.51 | $\|vvvvhhhhhhvv\rangle$ |
| 94.59 | $\|vvvvvvvvvvvv\rangle$ | 69.66 | $\|vvvvvvhhhhhh\rangle$ |

**Supplementary Table 6.** The coefficients $a_{4,i}\,(i=1,\cdots,16)$ for the ground states of system $\left|\Phi_g\right\rangle_4$

| | $g=10$ | $g=5$ | $g=2$ | $g=1$ | $g=0.9$ |
|---|---|---|---|---|---|
| $a_{4,1}$ | 0.7408 | 0.7508 | 0.7704 | 0.7811 | 0.7812 |
| $a_{4,2}$ | 0.2883 | 0.2754 | 0.2406 | 0.1945 | 0.1859 |
| $a_{4,4}$ | 0.1303 | 0.1353 | 0.1481 | 0.1641 | 0.1670 |
| $a_{4,8}$ | 0.0615 | 0.0695 | 0.0854 | 0.0942 | 0.0944 |
| $a_{4,16}$ | 0.0412 | 0.0609 | 0.1183 | 0.2037 | 0.2206 |

**Supplementary Table 7.** The coefficients $a_{4,i}\,(i=1,\cdots,16)$ for the MESs of system $\left|\Xi\right\rangle_4$

| | $g=0.2$ | $g=0.1$ |
|---|---|---|
| $a_{4,1}$ | 0 | -0.5386 |
| $a_{4,4}=a_{4,13}$ | -0.0104 | 0.5072 |
| $a_{4,6}=a_{4,11}$ | 0.5051 | 0.0060 |
| $a_{4,7}=a_{4,10}$ | -0.4947 | -0.0157 |
| $a_{4,16}$ | 0 | -0.4415 |

**Supplementary Table 8.** The coefficients $a_{8,i}(i=1,\cdots,256)$ for the ground states of system $\left|\Phi_g\right\rangle_8$

| | $g=10$ | $g=5$ | $g=2$ | $g=1$ | $g=0.9$ |
|---|---|---|---|---|---|
| $a_{8,1}$ | 0.5495 | 0.5663 | 0.6057 | 0.6340 | 0.6343 |
| $a_{8,2}$ | 0.2137 | 0.2071 | 0.1861 | 0.1513 | 0.1440 |
| $a_{8,4}$ | 0.0895 | 0.0875 | 0.0796 | 0.0636 | 0.0600 |
| $a_{8,6}$ | 0.0833 | 0.0765 | 0.0602 | 0.0412 | 0.0378 |
| $a_{8,8}$ | 0.0376 | 0.0373 | 0.0359 | 0.0312 | 0.0299 |
| $a_{8,16}$ | 0.0170 | 0.0184 | 0.0226 | 0.0286 | 0.0296 |
| $a_{8,18}$ | 0.0968 | 0.1026 | 0.1207 | 0.1536 | 0.1614 |
| $a_{8,20}$ | 0.0416 | 0.0449 | 0.0521 | 0.0573 | 0.0575 |
| $a_{8,22}$ | 0.0379 | 0.0385 | 0.0415 | 0.0465 | 0.0473 |
| $a_{8,24}$ | 0.0175 | 0.0193 | 0.0235 | 0.0264 | 0.0264 |
| $a_{8,28}$ | 0.0178 | 0.0193 | 0.0214 | 0.0201 | 0.0193 |
| $a_{8,32}$ | 0.0080 | 0.0095 | 0.0131 | 0.0160 | 0.0161 |
| $a_{8,52}$ | 0.0238 | 0.0323 | 0.0596 | 0.1088 | 0.1197 |
| $a_{8,56}$ | 0.0100 | 0.0132 | 0.0216 | 0.0309 | 0.0322 |
| $a_{8,64}$ | 0.0045 | 0.0063 | 0.0109 | 0.0156 | 0.0161 |
| $a_{8,86}$ | 0.0174 | 0.0201 | 0.0328 | 0.0680 | 0.0775 |
| $a_{8,88}$ | 0.0082 | 0.0101 | 0.0160 | 0.0247 | 0.0262 |
| $a_{8,96}$ | 0.0038 | 0.0049 | 0.0078 | 0.0100 | 0.0101 |
| $a_{8,120}$ | 0.0054 | 0.0084 | 0.0188 | 0.0407 | 0.0462 |
| $a_{8,128}$ | 0.0024 | 0.0039 | 0.0087 | 0.0160 | 0.0173 |
| $a_{8,256}$ | 0.0015 | 0.0030 | 0.0101 | 0.0303 | 0.0360 |

**Supplementary Table 9.** The entanglement entropies for the MES of toric code model with different strengths $g$ of external fields

| The entanglement entropies for MESs | | |
|---|---|---|
| $g$ | 8-spins system | 12-spins system |
| $g = 0.1$ | 1.3696 | 2.7606 |
| $g = 0.2$ | 1.3057 | 2.6981 |
| $g = 0.9$ | 0.5504 | 0.8735 |
| $g = 1$ | 0.4670 | 0.7352 |
| $g = 2$ | 0.1549 | 0.2379 |
| $g = 5$ | 0.0339 | 0.0515 |
| $g = 10$ | 0.0103 | 0.0155 |

**Supplementary Table 10**. The parameters for the FFT filters $\{FFT_1,\ldots,FFT_6\}$ and modulators $\{d_1,\ldots,d_5\}$ in Supplementary Figure 15 and their corresponding terms indicated in Supplementary Table 7.

| filtered frequency(MHz) | corresponding terms | amplitude modulation |
|---|---|---|
| $FFT_1$: 11.9 | $\lvert h_1h_2h_3h_4)$ | $d_1$ = -0.5386 |
| $FFT_2$:12.0 | $\lvert h_1h_2v_3v_4)$, $\lvert v_1v_2h_3h_4)$ | $d_2$ =0.5072 |
| $FFT_3$:2.0 | $\lvert h_1v_2h_3v_4)$ | $d_3$=0.0060 |
| $FFT_4$:1.8 | $\lvert v_1h_2v_3h_4)$ | |
| $FFT_5$: 1.9 | $\lvert v_1h_2h_3v_4)$, $\lvert h_1v_2v_3h_4)$ | $d_4$ = -0.0157 |
| $FFT_6$: 12.1 | $\lvert v_1v_2v_3v_4)$ | $d_5$= -0.4415 |

**Supplementary Table 11**. The parameters for the FFT filters $\{FFT_1,\ldots,FFT_9\}$ and modulators $\{d_1,\ldots,d_5\}$ in Supplementary Figure 16 and their corresponding terms in Supplementary Equation 28.

| filtered frequency(MHz) | corresponding terms | amplitude modulation |
|---|---|---|
| $FFT_1$: 11.9 | $|h_1h_2h_3h_4\rangle$ | $d_1$ = 0.7408 |
| $FFT_2$ :11.5 | $|h_1h_2v_3h_4\rangle$, $|h_1v_2h_3h_4\rangle$ | $d_2$ =0.2883 |
| $FFT_3$ :12.4 | $|h_1h_2h_3v_4\rangle$, $|v_1h_2h_3h_4\rangle$ | |
| $FFT_4$: 11.1 | $|h_1v_2v_3h_4\rangle$ | $d_3$ = 0.1303 |
| $FFT_5$: 12.0 | $|h_1h_2v_3v_4\rangle$ ,$|v_1v_2h_3h_4\rangle$ , $|h_1v_2h_3v_4\rangle$, $|v_1h_2v_3h_4\rangle$ | |
| $FFT_6$: 12.9 | $|v_1h_2h_3v_4\rangle$ | |
| $FFT_7$: 11.6 | $|h_1v_2v_3v_4\rangle$ ,$|v_1v_2v_3h_4\rangle$ | $d_4$ = 0.0615 |
| $FFT_8$: 12.5 | $|v_1h_2v_3v_4\rangle$ ,$|v_1v_2h_3v_4\rangle$ | |
| $FFT_9$: 12.1 | $|v_1v_2v_3v_4\rangle$ | $d_5$ = 0.0615 |

**Supplementary Table 12.** Distinct frequency components (MHz) selected by the bandpass filters $F_5$ and $F_6$ in the second stage in Supplementary Figure 17 and their corresponding terms.

| filter | $F_5$ (passband:6.8—18.0MHz) | | | | | | | |
|---|---|---|---|---|---|---|---|---|
| selected frequency | 7.18 | 10.17 | 9.92 | 12.91 | 9.53 | 12.52 | 12.27 | 15.26 |
| term | $|h_1h_2h_3h_4\rangle$ | $|h_1h_2h_3v_4\rangle$ | $|h_1h_2v_3h_4\rangle$ | $|h_1h_2v_3v_4\rangle$ | $|h_1v_2h_3h_4\rangle$ | $|h_1v_2h_3v_4\rangle$ | $|h_1v_2v_3h_4\rangle$ | $|h_1v_2v_3v_4\rangle$ |
| selected frequency | 9.45 | 12.44 | 12.19 | 15.18 | 11.8 | 14.79 | 14.54 | 17.53 |
| term | $|v_1h_2h_3h_4\rangle$ | $|v_1h_2h_3v_4\rangle$ | $|v_1h_2v_3h_4\rangle$ | $|v_1h_2v_3v_4\rangle$ | $|v_1v_2h_3h_4\rangle$ | $|v_1v_2h_3v_4\rangle$ | $|v_1v_2v_3h_4\rangle$ | $|v_1v_2v_3v_4\rangle$ |
| filter | $F_6$ (passband: 10.0—24.0MHz) | | | | | | | |
| selected frequency | 10.64 | 13.59 | 13.89 | 16.84 | 13.75 | 16.70 | 17.00 | 19.95 |

| | | | | | | | | |
|---|---|---|---|---|---|---|---|---|
| term | $\|h_5h_6h_7h_8)$ | $\|h_5h_6h_7v_8)$ | $\|h_5h_6v_7h_8)$ | $\|h_5h_6v_7v_8)$ | $\|h_5v_6h_7h_8)$ | $\|h_5v_6h_7v_8)$ | $\|h_5v_6v_7h_8)$ | $\|h_5v_6v_7v_8)$ |
| selected frequency | 13.74 | 16.69 | 16.99 | 19.94 | 16.85 | 19.80 | 20.10 | 23.05 |
| term | $\|v_5h_6h_7h_8)$ | $\|v_5h_6h_7v_8)$ | $\|v_5h_6v_7h_8)$ | $\|v_5h_6v_7v_8)$ | $\|v_5v_6h_7h_8)$ | $\|v_5v_6h_7v_8)$ | $\|v_5v_6v_7h_8)$ | $\|v_5v_6v_7v_8)$ |

**Supplementary Table 13**. Parameters of the 256 FFT filters and the modulators $\{d_1,\ldots,d_{21}\}$ at the bottom of Supplementary Figure 17 for the corresponding selected basis terms in Supplementary Table 8 for the case with $g=10$.

| Filtered frequency components (MHz) | Corresponding terms | Number of basis terms | Amplitude modulation |
|---|---|---|---|
| 17.82 | $\|hhhhhhhh)$ | 1 | $d_1$=0.5495 |
| 20.77, 21.07, …, 20.09 | $\|hhhhhhhv)$; $\|hhhhhhvh)$, $\cdots$, $\|vhhhhhhh)$ | 8 | $d_2$=0.2137 |
| 24.02, 24.18, …, 22.44 | $\|hhhhhhvv)$; $\|hhhhhvvh)$, $\cdots$, $\|vvhhhhhh)$ | 16 | $d_3$=0.0895 |
| 23.88, 24.17, …, 22.83 | $\|hhhhhvhv)$; $\|hhhhvhvh)$, $\cdots$, $\|vhvhhhhh)$ | 8 | $d_4$=0.0833 |
| 27.13, 27.12, …, 25.18 | $\|hhhhhvhv)$; $\|hhhhvhvh)$, $\cdots$, $\|vhvhhhhh)$ | 32 | $d_5$=0.0376 |
| 30.23, 30.27, …,28.13, 28.17 | $\|hhhhvvvv)$; $\|hhhvvvvh)$, $\cdots$, $\|vvvhhhhv)$; $\|vvvvhhhh)$ | 16 | $d_6$=0.0170 |
| 23.76, 23.81,23.28, 23.19 | $\|hhhvhhhv)$; $\|hhvhhhvh)$ $\|hvhhhvhh)$; $\|vhhhvhhh)$ | 4 | $d_7$=0.0968 |
| 27.01, 26.86, …,25.55, 25.54 | $\|hhhvhhvv)$; $\|hhhvvhhv)$, $\cdots$, $\|vvhhhvhh)$; $\|vvhhvhhh)$ | 16 | $d_8$=0.0416 |

| | | | |
|---|---|---|---|
| 26.87, 26.91, …,26.08, 25.93 | $\|hhhvhvhv\rangle$ ; $\|hhhvvhhv\rangle$ , ⋯ , $\|vhvhhhvh\rangle$ ; $\|vhvhvhhh\rangle$ | 8 | $d_9$ =0.0379 |
| 30.12, 29.97, …, 28.28 | $\|hhhvhvvv\rangle$ , $\|hhhvvvhv\rangle$ ⋯ , $\|vvvhvhhh\rangle$ | 32 | $d_{10}$ =0.0175 |
| 30.11, 29.87, …, 28.29 | $\|hhhvvhvv\rangle$ , $\|hhvhhvvv\rangle$ ⋯ , $\|vvvhhvhh\rangle$ | 16 | $d_{11}$ =0.0178 |
| 30.22, 32.97, …, 31.27 | $\|hhhvvvvv\rangle$ , $\|hhvhvvvv\rangle$ ⋯ , $\|vvvvvhhh\rangle$ | 32 | $d_{12}$ =0.0080 |
| 29.75, 29.27,29.13, 28.65 | $\|hhvvhhvv\rangle$ ; $\|hvvhhvvh\rangle$ , $\|hvvhhvvh\rangle$ ; $\|vvhhvvhh\rangle$ | 4 | $d_{13}$ =0.0238 |
| 32.86, 32.85, …, 31.39 | $\|hhvvhvvv\rangle$ , $\|hhvvvhvv\rangle$ ⋯ , $\|vvvhvvhh\rangle$ | 16 | $d_{14}$ =0.0100 |
| 35.96, 35.32, …, 34.38 | $\|hhvvvvvv\rangle$ , $\|hvvhvvvv\rangle$ ⋯ , $\|vvvvvvhh\rangle$ | 16 | $d_{15}$ =0.0045 |
| 29.22, 29.18 | $\|hvhvhvhv\rangle$ , $\|vhvhvhvh\rangle$ | 2 | $d_{16}$ =0.0174 |
| 32.47, 32.32, …, 31.53 | $\|hvhvhvvv\rangle$ , $\|hvhvvvhv\rangle$ ⋯ , $\|vvvhvhvh\rangle$ | 8 | $d_{17}$ =0.0082 |
| 35.57, 35.06, …, 34.52 | $\|hvhvvvvv\rangle$ , $\|hvvvvvhv\rangle$ ⋯ , $\|vvvvvhvh\rangle$ | 8 | $d_{18}$ =0.0038 |
| 35.21, 35.12, 34.59, 34.64 | $\|hvvvhvvv\rangle$ ; $\|vhvvvhvv\rangle$ , $\|vvhvvvhv\rangle$ ; $\|vvvhvvvh\rangle$ | 4 | $d_{19}$ =0.0054 |
| 38.31, 38.23, …, 37.63 | $\|hvvvvvvv\rangle$ , $\|vhvvvvvv\rangle$ ⋯ , $\|vvvvvvvh\rangle$ | 8 | $d_{20}$ =0.0024 |
| 40.58 | $\|vvvvvvvv\rangle$ | 1 | $d_{21}$ =0.0015 |

## Supplementary Note 1. Finding minimum entropy states (MESs) for square lattices with 8 and 12 spins

When the square lattice has 8 spins, the Hamiltonian for this system is

$$\begin{aligned} H_{0,8} = & -\sigma_x^1\sigma_x^2\sigma_x^6\sigma_x^5 - \sigma_z^2\sigma_z^3\sigma_z^7\sigma_z^6 - \sigma_x^3\sigma_x^4\sigma_x^8\sigma_x^7 - \sigma_z^4\sigma_z^1\sigma_z^5\sigma_z^8 \\ & -\sigma_z^5\sigma_z^6\sigma_z^2\sigma_z^1 - \sigma_x^6\sigma_x^7\sigma_x^3\sigma_x^2 - \sigma_z^7\sigma_z^8\sigma_z^4\sigma_z^3 - \sigma_x^8\sigma_x^5\sigma_x^1\sigma_x^4. \end{aligned} \tag{1}$$

The schematic representation has been addressed in Supplementary Figure 1.

There also exists four degenerated ground states for this Hamiltonian. Due to lengthy expressions of these four states, we only provide two of them ($\left|\varphi_{\mathrm{g},1}\right\rangle_8$ and $\left|\varphi_{\mathrm{g},2}\right\rangle_8$) below.

$$\begin{aligned} \left|\varphi_{\mathrm{g},1}\right\rangle_8 = \frac{1}{2\sqrt{2}}( & |00000000\rangle + |00110011\rangle + |01010101\rangle + |01100110\rangle \\ & + |10011001\rangle + |10101010\rangle + |11001100\rangle + |11111111\rangle), \end{aligned} \tag{2}$$

$$\begin{aligned} \left|\varphi_{\mathrm{g},2}\right\rangle_8 = \frac{1}{2\sqrt{2}}( & |00001111\rangle + |00111100\rangle + |01011010\rangle + |01101001\rangle \\ & - |10010110\rangle - |10100101\rangle - |11000011\rangle - |11110000\rangle). \end{aligned} \tag{3}$$

For the system with 8 spins, we can also define string operators as those in the system with 4 spins. Here, we generate two operators associated with two closed loops, one is along x-direction, and the other is along y-direction,

$$W_{\mathbf{e_x},8}^z(C) = \sigma_z^1\sigma_z^2\sigma_z^3\sigma_z^4,\ W_{\mathbf{e_y},8}^z(C) = \sigma_z^1\sigma_z^5. \tag{4}$$

The operators $W_{\mathbf{e_x},8}^z$ and $W_{\mathbf{e_y},8}^z$ represent the electric charge loop operators. After applying the string operator $W^z(C)$ to the degenerated ground states $\left|\varphi_{\mathrm{g},1}\right\rangle_8$ and $\left|\varphi_{\mathrm{g},2}\right\rangle_8$, we have relations as

$$W_{\mathbf{e_x},8}^z(C)\left|\varphi_{\mathrm{g},1}\right\rangle_8 = \left|\varphi_{\mathrm{g},1}\right\rangle_8,\ W_{\mathbf{e_y},8}^z(C)\left|\varphi_{\mathrm{g},1}\right\rangle_8 = \left|\varphi_{\mathrm{g},1}\right\rangle_8,\ W_{\mathbf{e_x},8}^z(C)\left|\varphi_{\mathrm{g},2}\right\rangle_8 = \left|\varphi_{\mathrm{g},2}\right\rangle_8,\ W_{\mathbf{e_y},8}^z(C)\left|\varphi_{\mathrm{g},2}\right\rangle_8 = -\left|\varphi_{\mathrm{g},2}\right\rangle_8. \tag{5}$$

Given the expressions of minimum entropy state (MES) in Ref. [1, 2], we take one MES for the system with 8 spins

$$|\psi\rangle_8 = \frac{1}{\sqrt{2}}\left(\left|\varphi_{\mathrm{g},1}\right\rangle_8 + \left|\varphi_{\mathrm{g},2}\right\rangle_8\right). \tag{6}$$

With $|\psi\rangle_8$, when separating the system into two subsystems as shown in Supplementary Figure 2, we can obtain the von Neumann entropy for the subsystem containing spins 1, 2, 3 and 4 as 2ln2.

When the square lattice has 12 spins, the Hamiltonian for this system is

$$\begin{aligned} H_{0,12} = & -\sigma_x^1\sigma_x^2\sigma_x^8\sigma_x^7 - \sigma_z^2\sigma_z^3\sigma_z^9\sigma_z^8 - \sigma_x^3\sigma_x^4\sigma_x^{10}\sigma_x^9 - \sigma_z^4\sigma_z^5\sigma_z^{11}\sigma_z^{10} - \sigma_x^5\sigma_x^6\sigma_x^{12}\sigma_x^{11} - \sigma_z^6\sigma_z^1\sigma_z^7\sigma_z^{12} \\ & -\sigma_z^1\sigma_z^2\sigma_z^8\sigma_z^7 - \sigma_x^2\sigma_x^3\sigma_x^9\sigma_x^8 - \sigma_z^3\sigma_z^4\sigma_z^{10}\sigma_z^9 - \sigma_x^4\sigma_x^5\sigma_x^{11}\sigma_x^{10} - \sigma_z^5\sigma_z^6\sigma_z^{12}\sigma_z^{11} - \sigma_x^6\sigma_x^1\sigma_x^7\sigma_x^{12}. \end{aligned} \tag{7}$$

The schematic representation has been addressed in Supplementary Figure 3.

There also exists four degenerated ground states for this Hamiltonian. Due to lengthy expressions of these four states, we only provide two of them ($\left|\varphi_{\mathrm{g},1}\right\rangle_{12}$ and $\left|\varphi_{\mathrm{g},2}\right\rangle_{12}$) below.

$$
\begin{aligned}
\left|\varphi_{g,1}\right\rangle_{12} = \frac{1}{4\sqrt{2}}(&|000000000000\rangle + |000011000011\rangle + |000101000101\rangle + |000110000110\rangle \\
&+|001001001001\rangle + |001010001010\rangle + |001100001100\rangle + |001111001111\rangle \\
&+|010001010001\rangle + |010010010010\rangle + |010100010100\rangle + |010111010111\rangle \\
&+|011000011000\rangle + |011011011011\rangle + |011101011101\rangle + |011110011110\rangle \\
&+|100001100001\rangle + |100010100010\rangle + |100100100100\rangle + |100111100111\rangle \\
&+|101000101000\rangle + |101011101011\rangle + |101101101101\rangle + |101110101110\rangle \\
&+|110000110000\rangle + |110011110011\rangle + |110101110101\rangle + |110110110110\rangle \\
&+|111001111001\rangle + |111010111010\rangle + |111100111100\rangle + |111111111111\rangle),
\end{aligned} \tag{8}
$$

$$
\begin{aligned}
\left|\varphi_{g,2}\right\rangle_{12} = \frac{1}{4\sqrt{2}}(&|000000111111\rangle + |000011111100\rangle + |000101111010\rangle + |000110111001\rangle \\
&+|001001110110\rangle + |001010110101\rangle + |001100110011\rangle + |001111110000\rangle \\
&+|010001101110\rangle + |010010101101\rangle + |010100101011\rangle + |010111101000\rangle \\
&+|011000100111\rangle + |011011100100\rangle + |011101100010\rangle + |011110100001\rangle \\
&+|100001011110\rangle + |100010011101\rangle + |100100011011\rangle + |100111011000\rangle \\
&+|101000010111\rangle + |101011010100\rangle + |101101010010\rangle + |101110010001\rangle \\
&+|110000001111\rangle + |110011001100\rangle + |110101001010\rangle + |110110001001\rangle \\
&+|111001000110\rangle + |111010000101\rangle + |111100000011\rangle + |111111000000\rangle).
\end{aligned} \tag{9}
$$

For the system with 12 spins, we can also define string operators as those in the system with 4 spins. Here, we generate two operators associated with two closed loops, one is along x-direction, and the other is along y-direction,

$$
W^z_{\mathbf{e}_x,12}(C) = \sigma_z^1\sigma_z^2\sigma_z^3\sigma_z^4\sigma_z^5\sigma_z^6,\ W^z_{\mathbf{e}_y,12}(C) = \sigma_z^1\sigma_z^7. \tag{10}
$$

The operators $W^z_{\mathbf{e}_x,12}$ and $W^z_{\mathbf{e}_y,12}$ represent the electric charge loop operators. After applying the string operator $W^z(C)$ to the degenerated ground states $\left|\varphi_{g,1}\right\rangle_{12}$ and $\left|\varphi_{g,2}\right\rangle_{12}$, we have relations as

$$
W^z_{\mathbf{e}_x,12}(C)\left|\varphi_{g,1}\right\rangle_{12} = \left|\varphi_{g,1}\right\rangle_{12},\ W^z_{\mathbf{e}_y,12}(C)\left|\varphi_{g,1}\right\rangle_{12} = \left|\varphi_{g,1}\right\rangle_{12},\ W^z_{\mathbf{e}_x,12}(C)\left|\varphi_{g,2}\right\rangle_{12} = \left|\varphi_{g,2}\right\rangle_{12},\ W^z_{\mathbf{e}_y,12}(C)\left|\varphi_{g,2}\right\rangle_{12} = -\left|\varphi_{g,2}\right\rangle_{12}. \tag{11}
$$

Given the expressions of MES in Ref. [1, 2], we take one MES for the system with 12 spins as

$$
|\psi\rangle_{12} = \frac{1}{\sqrt{2}}\left(\left|\varphi_{g,1}\right\rangle_{12} + \left|\varphi_{g,2}\right\rangle_{12}\right). \tag{12}
$$

With $|\psi\rangle_{12}$, when separating the system into two subsystems as shown in Supplementary Figure 4, we can obtain the von Neumann entropy for the subsystem containing spins 1, 2, 3, 4, 5 and 6 as 4ln2.

## Supplementary Note 2. Finding MESs for toric code models with 8 and 12 spins

Here, we present the entanglement entropy for real toric code model, with the spin occupying at the bond of lattice.

We only present the entropies for such model with 8 spins and 12 spins. The Hamiltonian for such model with 8 spins is

$$\begin{aligned} H^{\mathrm{tc}}{}_{0,8} = &-\sigma_x^1\sigma_x^3\sigma_x^2\sigma_x^7 - \sigma_z^1\sigma_z^3\sigma_z^4\sigma_z^5 - \sigma_x^5\sigma_x^3\sigma_x^7\sigma_x^6 - \sigma_z^5\sigma_z^8\sigma_z^7\sigma_z^1 \\ &-\sigma_z^2\sigma_z^3\sigma_z^4\sigma_z^6 - \sigma_x^1\sigma_x^2\sigma_x^4\sigma_x^8 - \sigma_z^6\sigma_z^7\sigma_z^8\sigma_z^2 - \sigma_x^4\sigma_x^5\sigma_x^6\sigma_x^8, \end{aligned} \tag{13}$$

which is the smallest toric code model exhibiting the nontrivial entanglement entropy. The schematic representation is addressed in Supplementary Figure 5.

When we have obtained the four degenerated ground states for this model, we look for the MES of such model. We find that the MES has the similar form as in the equivalent square lattice. The von Neumann entropy for this MES is 2ln2.

When the system contains 12 spins, the Hamiltonian for this system is

$$\begin{aligned} H^{\mathrm{tc}}{}_{0,12} = &-\sigma_x^1\sigma_x^{10}\sigma_x^4\sigma_x^3 - \sigma_z^1\sigma_z^{10}\sigma_z^{11}\sigma_z^7 - \sigma_x^4\sigma_x^7\sigma_x^{10}\sigma_x^9 - \sigma_z^1\sigma_z^4\sigma_z^5\sigma_z^7 - \sigma_x^1\sigma_x^2\sigma_x^5\sigma_x^{11} - \sigma_z^2\sigma_z^8\sigma_z^{11}\sigma_z^{12} \\ &-\sigma_z^2\sigma_z^5\sigma_z^6\sigma_z^8 - \sigma_x^5\sigma_x^7\sigma_x^8\sigma_x^{11} - \sigma_z^3\sigma_z^4\sigma_z^6\sigma_z^9 - \sigma_x^2\sigma_x^3\sigma_x^6\sigma_x^{12} - \sigma_z^9\sigma_z^3\sigma_z^{10}\sigma_z^{12} - \sigma_x^6\sigma_x^8\sigma_x^9\sigma_x^{12}. \end{aligned} \tag{14}$$

The schematic representation for this model is addressed in Supplementary Figure 6.

When we have obtained the four degenerated ground states for this model, we look for the MES of such model. We find that the MES has the similar form as in the equivalent square lattice. The von Neumann entropy for this MES is 4ln2. So we can extract the topological entanglement entropy (TEE) from these entanglement entropies for the toric code model with 8 and 12 spins (Supplementary Figure 7).

In our study of toric code model, since the periodic boundary condition is chosen, there exists two disconnected boundaries between two subsystems (Supplementary Figures 5 and 6). Therefore, the entropies of one subsystem can be expressed as $S_{\mathrm{A}} = \alpha L_{\mathrm{x}} - m\gamma + \ldots$ with $m = 2$. The parameter $L_{\mathrm{x}}$ represents the length for one boundary. From these entropies, we can extract the TEE value as $\gamma = \ln 2$, which agrees with that extracted from the square lattice.

## Supplementary Note 3. The detailed descriptions on the experiments for 4-cebit analogies of MESs

**Experimental details for constructing 4-cebit analogies of MESs.** Here we show the details of the process for constructing and measuring the desired 4-cebit classical minimal entropy states (CMES) as shown in Fig. 3 of main text. In Fig. 3(a), the microwave signals with vertical and horizontal polarizations at the frequency 17 GHZ are produced by a dual-polarized transmitting antenna and received by *n* dual-polarized antennas. Each dual-polarized receiving antenna includes 2 channels $\{h_i, v_i\}$, and the induced electric voltage signals in the $h_i$ ( $v_i$ ) channel are denoted as $P_{h_i}$ ( $P_{v_i}$ ) $(i = 1, 2, \ldots, n)$. After a 2*n*-channel microwave receiver, these multiplex signals are down-converted from an RF frequency to an intermediate frequency (IF) of 140MHz. Subsequently, all 2*n* signals can be digitalized by using an analog-to-digital converter (ADC), at which point all further signal processing is done using digital signal processing (DSP) techniques as shown in Fig. 3(b).

In Fig. 3(b), we first arrange eight signals in the set of channels $\{ h_1, v_1, h_2, v_2, h_3, v_3, h_4, v_4 \}$ fed into the DSP module to their respective frequencies using a digital down-converter (DDC) as shown in Fig. 3(b). After tuning the

same initial phases for all signals, a pair of signals output from the $\{h_i, v_i\}$ channel of the DDC can be expressed assembly as

$$\{B_{h_i}(t), B_{v_i}(t)\} = \{B_{h_i}\exp(-\mathrm{i}\omega_{h_i}t), B_{v_i}\exp(-\mathrm{i}\omega_{v_i}t)\}, \tag{15}$$

where $B_{h_i}(B_{v_i}) \propto P_{h_i}(P_{v_i})$, $t = n\Delta T$ with $\Delta T$ being the sampling time interval, and $\omega_{h_i(v_i)}(= 2\pi f_{h_i(v_i)})$ is the corresponding down-converted angular frequency set as

$$\begin{aligned} &f_{h_1} = 1.4\text{MHz}, f_{v_1} = 1.7\text{MHz}, f_{h_2} = 2.6\text{MHz}, f_{v_2} = 2.3\text{MHz}, \\ &f_{h_3} = 3.7\text{MHz}, f_{v_3} = 3.3\text{MHz}, f_{h_4} = 4.3\text{MHz}, f_{v_4} = 4.7\text{MHz}. \end{aligned} \tag{16}$$

In the experiment, the classical analogy of the projective measurement can be realized by subsequent modulations of the $\{B_{h_i}(t), B_{v_i}(t)\}$ in the projection part (PROJ). If the projective measurement basis for the *i*th signal pair is set as $|\mathbf{e}_{\mathrm{m}_i}) = (c_{h_i}, c_{v_i})^{\mathrm{T}}$, then the resultant signal $S_i(t)$ of the projection operation is defined as the sum of the signals in the channel $\{h_i, v_i\}$ after they are multiplied by the projection coefficient $(c^*_{h_i}, c^*_{v_i})$ set in the PROJ as

$$\begin{aligned} S_i(t) &= c^*_{h_i}B_{h_i}(t) + c^*_{v_i}B_{v_i}(t) \\ &\propto c^*_{h_i}P_{h_i}\exp(-\mathrm{i}\omega_{h_i}t) + c^*_{v_i}P_{v_i}\exp(-\mathrm{i}\omega_{v_i}t) \\ &=(\mathbf{e}_{\mathrm{m}_i}|h_i)P_{h_i}\exp(-\mathrm{i}\omega_{h_i}t) + (\mathbf{e}_{\mathrm{m}_i}|v_i)P_{v_i}\exp(-\mathrm{i}\omega_{v_i}t), \end{aligned} \tag{17}$$

where $(\mathbf{e}_{\mathrm{m}_i}| = (c^*_{h_i}, c^*_{v_i})$ is the conjugate transpose for $|\mathbf{e}_{\mathrm{m}_i}) = (c_{h_i}, c_{v_i})^{\mathrm{T}}$, and $|h_i) = (1,0)^{\mathrm{T}}_i$ ($|v_i) = (0,1)^{\mathrm{T}}_i$) represents the horizontal (vertical) unit amplitude signal in the $h_i$ ($v_i$) channel. Such a unit of the PROJ is highlighted in the inset of Fig. 3(b). Based on the basis signal $|h_i)$ and $|v_i)$, we can organize any designated composite signal in the DSP module into the form of a Jones vector [3] as proposed in the optical experiment. For example, Supplementary Equation 17 can be expressed as $(\mathbf{e}_{\mathrm{m}_i}|\left\{B_{h_i}|h_i) + B_{v_i}|v_i)\right\}$. Moreover, here we follow the proposed concept of cebit [4, 5] to name the vector form of a signal pair as the classical counterpart of a single-qubit quantum state, and adopt the parentheses notation (parent $(|$ and thesis $|)$) to represent it as $(B_{h_i}, B_{v_i})^{\mathrm{T}}$. The cebits constitute an inner product space where the inner product is given by parentheses $(|)$.

After the projection measurement process in the PROJ, the output signals $\{S_1(t), S_2(t)\}$ are sent to a multiplier ($\times$) and then a digital filter $F_{12}$, where the resulting product signal is selected at the sum frequency $f_{h_1} + f_{h_2} = f_{v_1} + f_{v_2} = 4.0\text{MHz}$. Note this signal denoted $S_{12}(t)$ contains the $|h_1)|h_2)$ and $|v_1)|v_2)$ components, which are prepared to participate in simulating the corresponding quantum state $|\psi\rangle_4$. Similarly, the signals $\{S_3(t), S_4(t)\}$ are multiplied and filtered at $f_{h_3} + f_{h_4} = f_{v_3} + f_{v_4} = 8.0\text{MHz}$ by $F_{34}$, yielding a signal amplitude $S_{34}(t)$ which contains two contributions, i.e., the $|h_3)|h_4)$ and $|v_3)|v_4)$ components at 8.0MHz.

Now, the two product signals, $S_{12}(t)$ and $S_{34}(t)$, continue to go through a multiplier followed by an FFT-based digital filter. Then, only one signal component containing four indistinguishable contributions $|h_1)|h_2)|h_3)|h_4)$, $|h_1)|h_2)|v_3)|v_4)$, $|v_1)|v_2)|h_3)|h_4)$ and $|v_1)|v_2)|v_3)|v_4)$ is selected with the desired frequency $\Omega$ as

$$\begin{aligned} \Omega &= f_{h_1} + f_{h_2} + f_{h_3} + f_{h_4} = f_{h_1} + f_{h_2} + f_{v_3} + f_{v_4} \\ &= f_{v_1} + f_{v_2} + f_{h_3} + f_{h_4} = f_{v_1} + f_{v_2} + f_{v_3} + f_{v_4} = 12.0\text{MHz}, \end{aligned} \tag{18}$$

and the corresponding complex amplitude $A_\Omega$ of this final filtered signal is expressed as

$$\begin{aligned} A_\Omega \propto & P_1 P_2 P_3 P_4 \\ & \times[(\mathbf{e}_{m_1}|h_1)(\mathbf{e}_{m_2}|h_2)(\mathbf{e}_{m_3}|h_3)(\mathbf{e}_{m_4}|h_4)+(\mathbf{e}_{m_1}|h_1)(\mathbf{e}_{m_2}|h_2)(\mathbf{e}_{m_3}|v_3)(\mathbf{e}_{m_4}|v_4)+ \\ & (\mathbf{e}_{m_1}|v_1)(\mathbf{e}_{m_2}|v_2)(\mathbf{e}_{m_3}|h_3)(\mathbf{e}_{m_4}|h_4)+(\mathbf{e}_{m_1}|v_1)(\mathbf{e}_{m_2}|v_2)(\mathbf{e}_{m_3}|v_3)(\mathbf{e}_{m_4}|v_4)]. \end{aligned} \tag{19}$$

under the far-field approximation condition $P_{h_i}=P_{v_i}=P_i\ (i=1,2,3,4)$, where $(\mathbf{e}_{m_1}|(\mathbf{e}_{m_2}|(\mathbf{e}_{m_3}|(\mathbf{e}_{m_4}|$ is the conjugate transpose of the column vector $|\mathbf{e}_{m_1})|\mathbf{e}_{m_2})|\mathbf{e}_{m_3})|\mathbf{e}_{m_4})$ as the projective measurement basis arranged in the PROJ. This complex amplitude $A_\Omega$ corresponds to the projection probability amplitude of the four-qubit state $|\psi\rangle_4$ onto a basis setting $|\mathbf{e}_{m_1})|\mathbf{e}_{m_2})|\mathbf{e}_{m_3})|\mathbf{e}_{m_4})$.

**Characterization of the 4-cebit CMES.** Considering the traditional method to characterize the quantum states produced in the photonic experiments [6-8], we can verify the quality of our constructed 4-cebit CMES in a similar way.

In the quantum information field, quantum state tomography is a widely accepted approach to characterize the quality of the density matrix for a prepared quantum state. Similarly, we can also consider a Hermitian and semi-definite matrix $\rho_4^{\mathrm{cl}}$ associated with the form in Eq. (6) of main text, which can be estimated using a proposed similar state tomography based on a set of experimental measurement outcomes. Therefore, cube measurement sets [9] are taken for the basis $|\mathbf{e}_{m_i})\ (i=1,2,3,4)$, where each basis $|\mathbf{e}_{m_i})$ can take one of the set $\{|h),|v),|+),|-),|l),|r)\}$. By measuring the projections of all 1296 bases and using a simple state reconstruction algorithm [10] with an efficient maximum-likelihood technique [11], we can obtain the experimental 4-cebit classical analogy of density matrix $\rho_4^{\mathrm{cl}}$.

In our experimental simulation, the needed experimental outcomes are the normalized signal intensities identified as probabilities of projections (the modulus squared of $A_\Omega$ in Supplementary Equation 19) compared with ideal values. These experimental observations can be interpreted in terms of the constructed state form of $|\psi_4^{\mathrm{cl}})$ in Eq. (6) of main text, which is an equal superposition of four terms $|h_1)|h_2)|h_3)|h_4)$, $|h_1)|h_2)|v_3)|v_4)$, $|v_1)|v_2)|h_3)|h_4)$ and $|v_1)|v_2)|v_3)|v_4)$. Such a form is capable of producing an interference effect as expected. For example, the projection of $|\psi_4^{\mathrm{cl}})$ onto the measurement basis $|+_1)|+_2)|+_3)|-_4)$ is 0, which indicates a destructive interference between the four superposed terms. By contrast, ideally this projection probability can reach 0.25 in the basis $|+_1)|+_2)|+_3)|+_4)$, revealing the occurrence of a constructive interference result. Since this classical signal protocol can well reproduce the probability distribution predicted in quantum theory, we can observe that the reconstructed density matrices in Supplementary Figure 8 (a) and (b) agree well with the predicted quantum state, which can reach a high fidelity of $0.9977 \pm 0.0009$.

## Supplementary Note 4. The detailed descriptions on the experiments for 8- and 12-cebit CMESs

**Experimental construction of 8-cebit CMES.** We can construct the 8-cebit CMES $|\psi_8^{\mathrm{cl}})$ denoted as

$$\begin{aligned} |\psi_8^{\mathrm{cl}})= & =\frac{1}{4}[|hhhhhhhh)+|hhvvhhvv)+|hvhvhvhv)+|hvvhhvvh)+ \\ & |vhhvvhhv)+|vhvhvhvh)+|vvhhvvhh)+|vvvvvvvv)+ \\ & |hhhhvvvv)+|hhvvvvhh)+|hvhvvhvh)+|hvvhvhhv)+ \\ & |vhhvhvvh)+|vhvhhvhv)+|vvhhhhvv)+|vvvvhhhh)], \end{aligned} \tag{20}$$

which corresponds to the quantum state $|\psi\rangle_8$. The designed circuit in the digital signal processing (DSP) module is presented in Supplementary Figure 9.

First we set the down-converted frequencies of the 16 signals in channels $\{h_1, v_1, \cdots h_8, v_8\}$ through the DDC in Supplementary Figure 9 to be

$$\begin{aligned} &f_{h_1} = 1.54\text{MHz}, \quad f_{v_1} = 3.81\text{MHz}, \quad f_{h_2} = 1.71\text{MHz}, \quad f_{v_2} = 4.06\text{MHz}, \\ &f_{h_3} = 1.90\text{MHz}, \quad f_{v_3} = 4.64\text{MHz}, \quad f_{h_4} = 2.03\text{MHz}, \quad f_{v_4} = 5.02\text{MHz}, \\ &f_{h_5} = 2.24\text{MHz}, \quad f_{v_5} = 5.34\text{MHz}, \quad f_{h_6} = 2.51\text{MHz}, \quad f_{v_6} = 5.62\text{MHz}, \\ &f_{h_7} = 2.73\text{MHz}, \quad f_{v_7} = 5.98\text{MHz}, \quad f_{h_8} = 3.16\text{MHz}, \quad f_{v_8} = 6.11\text{MHz}. \end{aligned} \tag{21}$$

As shown in Supplementary Figure 9, these eight signals $\{B_{h_1}, B_{v_1}, \cdots, B_{h_4}, B_{v_4}\}$ go through the PROJ and then enter a stage-by-stage mixing and filtering process.

In the first stage, the recombined signals $S_1$ and $S_2$ are processed by the multiplier $M_1$ and then a bandpass filter $F_1$ (passband: 3.0—8.0MHz), which would select 4 distinct frequencies 3.25, 5.60, 5.52, and 7.87MHz indicating the four terms $|h_1h_2)$, $|h_1v_2)$, $|v_1h_2)$, and $|v_1v_2)$ as shown in Supplementary Table 1 to be recombined by an adder. Similarly, the signals $S_3$ and $S_4$ go through the multiplier $M_2$, a bandpass filter $F_2$ (passband: 3.5—10.0MHz), and an adder. Then, these two summed signals $S_{12}$ and $S_{34}$ are sent into the second stage, processed by a multiplier $M_5$ for mixing and three filter $F_5$ (7.18MHz), $F_6$ (passband: 11.0—13.5MHz) and $F_7$ (17.53MHz) which together select eight desired sum-frequency components $\{|h_1h_2h_3h_4), \ldots, |v_1v_2v_3v_4)\}$ as listed in Supplementary Table 1 to be summed by an adder.

At the same time, the other eight signals $\{B_{h_5}, B_{v_5}, \cdots, B_{h_8}, B_{v_8}\}$ are processed by the corresponding first (the multipliers $M_3$, $M_4$; filters $F_3$, $F_4$) and second stages (the multipliers $M_6$; filters $F_8$, $F_9$ and $F_{10}$), and finally eight desired sum-frequency components are selected as listed in Supplementary Table 2 to be summed by an adder.

In the following, the sum signal of eight components $\{|h_1h_2h_3h_4), \ldots, |v_1v_2v_3v_4)\}$ in Supplementary Table 1 denoted as $S_{1-4}$ and the sum signal of eight components $\{|h_5h_6h_7h_8), \ldots, |v_5v_6v_7v_8)\}$ in Supplementary Table 2 represented as $S_{5-8}$ are processed by a multiplier $M_7$ in Supplementary Figure 9. Then the combined signal passes through a collection of FFT-based digital filters $\{FFT_1, \ldots, FFT_{16}\}$ where 16 amplitudes $\{A_{\omega_1}, A_{\omega_2}, \ldots, A_{\omega_{16}}\}$ of the desired frequency components are selected respectively corresponding to those 16 superposed terms in Supplementary Equation 20. The filtered frequencies and the corresponding terms are listed in Supplementary Table 3.

Finally, the composite signal representing the projection of the 8-cebit CMES described in Supplementary Equation 21 onto a measurement basis can be recorded. The amplitude of this composite signal is expressed as $A = \sum_{i=1}^{16} A_{\omega_i}$. Similar as the discussion in 4-cebit CMES, here with the signal $A$ from different microwave measurement basis, we can characterize the quality of prepared 8-cebit CMES based on the state tomography method. The details of characterization of the 8-cebit CMESs will be presented in Supplementary Note 5.

**Experimental construction of 12-cebit CMES.** We can construct the 12-cebit CMES $|\psi_{12}^{\text{cl}})$ as

$$\begin{aligned} |\psi_{12}^{\text{cl}}) = &= \frac{1}{4\sqrt{2}}[|hhhhhhhhhhhh) + |hhhhvvhhhhvv) + \ldots + |vvvvhhvvvvhh) + |vvvvvvvvvvvv) + \\ &|hhhhhhvvvvvv) + |hhhhvvvvvvhh) + \ldots + |vvvvhhhhhhvv) + |vvvvvvhhhhhh)], \end{aligned} \tag{22}$$

which corresponds to the MES $|\psi\rangle_{12}$. The designed circuit in the digital signal processing (DSP) module is

presented in Supplementary Figure 10.

We set the down-converted frequencies of the 24 signals in channels $\{h_1, v_1, \cdots h_{12}, v_{12}\}$ through the DDC in Supplementary Figure 10 as

$$\begin{aligned}
&f_{h_1}=1.54\text{MHz},\ f_{v_1}=6.08\text{MHz},\ f_{h_2}=1.81\text{MHz},\ f_{v_2}=6.41\text{MHz},\\
&f_{h_3}=2.23\text{MHz},\ f_{v_3}=6.75\text{MHz},\ f_{h_4}=2.61\text{MHz},\ f_{v_4}=7.02\text{MHz},\\
&f_{h_5}=2.83\text{MHz},\ f_{v_5}=7.39\text{MHz},\ f_{h_6}=3.16\text{MHz},\ f_{v_6}=7.66\text{MHz},\\
&f_{h_7}=3.51\text{MHz},\ f_{v_7}=8.02\text{MHz},\ f_{h_8}=4.06\text{MHz},\ f_{v_8}=8.41\text{MHz},\\
&f_{h_9}=4.64\text{MHz},\ f_{v_9}=8.75\text{MHz},\ f_{h_{10}}=5.02\text{MHz},\ f_{v_{10}}=9.07\text{MHz},\\
&f_{h_{11}}=5.42\text{MHz},\ f_{v_{11}}=9.33\text{MHz},\ f_{h_{12}}=5.70\text{MHz},\ f_{v_{12}}=9.70\text{MHz}.
\end{aligned} \tag{23}$$

As shown in Supplementary Figure 10, these 24 signals $\{B_{h_1}, B_{v_1}, \cdots, B_{h_{12}}, B_{v_{12}}\}$ go through the PROJ and then enter a stage-by-stage mixing and filtering process.

Note the processes designed in the first and second stages are similar to those in Supplementary Figure 9. For simplicity, here we only list the parameters of these bandpass filters $\{F_1, F_2, \ldots, F_6\}$ and $\{F_7, F_8, F_9\}$ in Supplementary Table 4 (note $F_9$ contains 16 filtered frequency points), and the resultant summed product signals denoted as $S_{1-4}$, $S_{5-8}$ and those 16 signals indicating the terms $\{|h_9h_{10}h_{11}h_{12}), \ldots, |v_9v_{10}v_{11}v_{12})\}$ are sent into the third stage for constructing the 12-cebit analogy state $|\psi_{12}^{\text{cl}})$.

Then, we focus on the signal processing in the third stage. The mixed signal resulting from the mixing of two former signals $S_{1-4}$ and $S_{5-8}$ by a multiplier $M_{10}$ is processed by a collection of filters $\{F_{10}, F_{11}, \ldots, F_{72}, F_{73}\}$. Notice that the 64 superposed terms in the target 12-cebit analogy state in Supplementary Equation 22 can be classified into 16 groups by the form of the cebits 9, 10, 11, 12, and thus we can recombine the signals entering the third stage by filtering and mixing to represent each term in the state. For example, the four components $\{|h_1h_2h_3h_4h_5h_6h_7h_8), |v_1v_2h_3h_4h_5h_6v_7v_8), |h_1h_2v_3v_4v_5v_6v_7v_8), |v_1v_2v_3v_4v_5v_6h_7h_8)\}$ can be put into one group since they own the same joint part of $|h_9h_{10}h_{11}h_{12})$ as shown in Supplementary Equation 22, and thus are selected by the first four filters $\{F_{10}, F_{11}, F_{12}, F_{13}\}$ at 21.75 MHz, 39.75 MHz, 48.60 MHz and 48.88MHz, respectively. Then, these four signals are added into a summed signal denoted as $S_{1-8}^{(hhhh)}$, which is multiplied by another signal indicating the $|h_9h_{10}h_{11}h_{12})$ component at 20.78MHz. Finally, such obtained mixed signal the four associated FFT-based filters $\{FFT_1, FFT_2, FFT_3, FFT_4\}$, where four desired frequency signals $\{A_{\omega_1}, A_{\omega_2}, A_{\omega_3}, A_{\omega_4}\}$ are selected corresponding to the four terms $\{|h_1h_2h_3h_4h_5h_6h_7h_8h_9h_{10}h_{11}h_{12})$, $|v_1v_2h_3h_4h_5h_6v_7v_8h_9h_{10}h_{11}h_{12})$, $|h_1h_2v_3v_4v_5v_6v_7v_8h_9h_{10}h_{11}h_{12})$, $|v_1v_2v_3v_4v_5v_6h_7h_8h_9h_{10}h_{11}h_{12})\}$, respectively as listed in Supplementary Table 5. In a similar way, we implement signal processing for obtaining the analogies of other superposed terms in Supplementary Equation 22, and the filtered frequencies for all desired corresponding terms are listed in Supplementary Table 5.

Thus, the composite signal representing the projection of the 12-cebit CMES described in Supplementary Equation 22 onto a measurement basis can be recorded. The amplitude of this composite signal is expressed as $A = \sum_{i=1}^{64} A_{\omega_i}$. Here with the signal $A$ from different microwave measurement basis, we can then characterize the quality of prepared 12-cebit CMES based on the state tomography method similar to that for the 8-cebit cases as presented in Supplementary Note 5.

## Supplementary Note 5. The compressed sensing method for state tomography

Compressed sensing concerns the problem of recovering structured signals (e.g., sparse signals and low rank matrices) from a small number of measurements [12-14]. When the desired density matrices are low-rank, it has been shown that one can stably reconstruct these matrices from highly incomplete Pauli measurements via some convex recovery procedures [15-17].

Let $\Gamma = \otimes_{j=1}^{n} \sigma_j$ be an *n*-qubit composite Pauli matrix with $\sigma_j \in \{I, \sigma_x, \sigma_y, \sigma_z\}$. For the *d*-dimensional Hilbert space, the corresponding Pauli basis has $d^2$ composite Pauli matrices denoted by $\{\Gamma_k\}_{k=1}^{d^2}$.In the quantum experiment, we only randomly choose *m* ($m \ll d^2$) composite Pauli matrices denoted as $\{\Gamma(A_i)\}_{i=1}^{m}$ ($A_1, \ldots, A_m \in [1, d^2]$) and measure their expectation values $\{b(A_i)\}_{i=1}^{m}$. For each measurement setting, $2^n$ outcomes are recorded, from which the corresponding Pauli expectation values can be computed. The theory of compressed sensing guarantees that $m = O(rd)$ measurements suffice to recover the desired density matrices with high probability, where *r* is the rank of the desired matrices. Then we can recover the density matrices $\rho$ by solving the following convex optimization problem:

$$\min \|\rho\|_{\mathrm{tr}} \quad , \text{s.t.} \ \sum_{i=1}^{m} [\mathrm{tr}(\Gamma(A_i)\rho) - b(A_i)]^2 \le \varepsilon \quad , \tag{24}$$

where $\|\cdot\|_{\mathrm{tr}}$ denotes the nuclear norm of a matrix and $\varepsilon$ represents a parameter indicating the estimating errors of the experimental data.

For the 8-cebit analogy cases, we choose *m*=6000( $m/d^2 \approx 0.1$ ) composite Pauli matrices randomly, and calculate their expectation values using the normalized projection values obtained from Supplementary Figure 9. Substituting these *m* values denoted as $\{b(A_i)\}_{i=1}^{m}$ into Supplementary Equation 24, we can estimate the density matrix $\rho_8^{\mathrm{cl}}$ by employing the semidefinite programming (SDP) solver SDPT3 included in the MATLAB toolbox CVX [18]. That is, we solved the nuclear norm minimization problem with an additional constraint that the decision variable be positive definite. The recovered solution from this technique is approximately low-rank. Moreover, whenever the trace of the resulting estimate is less than 1, we renormalize the state , $\rho_8^{\mathrm{cl}} / \mathrm{Tr}(\rho_8^{\mathrm{cl}}) \mapsto \rho_8^{\mathrm{cl}}$ [16]. The fidelity of the experimental obtained 8-cebit CMES is $0.9855 \pm 0.0045$.

For the reconstruction of the 12-cebit CMES, the dimensionality is $d$ =$2^{12}$=4096 and we set the number of random selected Pauli matrices ( $\Gamma = \otimes_{j=1}^{14} \sigma_j$ ) employed in the state tomography to be $5 \times 10^4$ (comparable to $d \log d$ ). In this case, we solve this optimization problem (Supplementary Equation 24) through distributed computing. We obtain the reconstructed density matrix denoted as $\rho_{14}^{\mathrm{cl}}$ with a high fidelity of $0.9683 \pm 0.0056$.

## Supplementary Note 6. The construction of MESs for square lattices with symmetrically applied fields

In our study, when an external field is added on the system, the Hamiltonian of the square lattice changes to

$$H_{\mathrm{t}} = H_0 + H_1, \tag{25}$$

where $H_0$ describes the square lattice without external fields. The Hamiltonian $H_1$ represents the external fields and takes the form as

$$H_1 = -g\sum_{i=1}^{n}(\sigma_x^i + \sigma_z^i)\,. \qquad (26)$$

The strength $g$ represents the strength of symmetrically external fields. The subscript $i$ is chosen for all the spins on the lattice. The detailed forms of the square lattice with 4 spins as

$$H_{t,4} = -2(\sigma_x^1\sigma_x^2\sigma_x^3\sigma_x^4 + \sigma_z^1\sigma_z^2\sigma_z^3\sigma_z^4) - g\left(\sigma_x^1 + \sigma_x^2 + \sigma_x^3 + \sigma_x^4 + \sigma_z^1 + \sigma_z^2 + \sigma_z^3 + \sigma_z^4\right), \qquad (27)$$

The schematic representation is shown in Supplementary Figure 11. We express the states with 4 spins as

$$|\Phi\rangle_4 = a_{4,1}|0000\rangle + a_{4,2}|0001\rangle + a_{4,3}|0010\rangle + \cdots + a_{4,16}|1111\rangle, \qquad (28)$$

which can be written as

$$|\Phi\rangle_4 = \begin{pmatrix} a_{4,1} \\ a_{4,2} \\ a_{4,3} \\ \vdots \\ a_{4,16} \end{pmatrix}. \qquad (29)$$

When the system belongs to topologically trivial phase ( $g > 0.34$ ), there are no nearly degenerated ground states and the topological properties can be revealed from its unique ground state. In order to describe the topological properties of system with external fields, we need to obtain the corresponding ground states for systems. Since there are no analytic forms of ground states for the system with external fields, we numerically obtain the ground states of system ( $|\Phi_g\rangle_4$ ) and list as below. When the system has 4 spins and $g \geq 0.9$, we find that some coefficients in the ground state $|\Phi_g\rangle_4$ are same, that is

(1) $a_{4,1}$,

(2) $a_{4,2} = a_{4,3} = a_{4,5} = a_{4,9}$,

(3) $a_{4,4} = a_{4,6} = a_{4,7} = a_{4,10} = a_{4,11} = a_{4,13}$,

(4) $a_{4,8} = a_{4,12} = a_{4,14} = a_{4,15}$,

(5) $a_{4,16}$. (30)

The coefficients $a_{4,i}(i = 1,\cdots,16)$ for the ground states of system $|\Phi_g\rangle_4$ with different strengths $g$ of external fields are shown in Supplementary Table 6.

When the system has 4 spins and $g \leq 0.34$, the system belongs to the $Z_2$ phase and there exist four nearly degenerated ground states. By using the numerical diagonalization to the system Hamiltonian, we can obtain such four nearly degenerated ground states. We do the linear combination for these four nearly degenerated ground states and obtain the MES. Here we present the coefficients $a_{4,i}(i = 1,\cdots,16)$ of each MES as

$g = 0.1$,

$$\left|\Xi\right\rangle_4 = 0.4\left|\phi_1\right\rangle - 0.7\left|\phi_2\right\rangle - 0.1\left|\phi_3\right\rangle + 0.5831\left|\phi_4\right\rangle = \begin{pmatrix} a_{4,1} \\ a_{4,2} \\ a_{4,3} \\ \vdots \\ a_{4,16} \end{pmatrix}; \tag{31}$$

$g = 0.2$,

$$\left|\Xi\right\rangle_4 = 0.95\left|\phi_3\right\rangle + 0.3122\left|\phi_4\right\rangle = \begin{pmatrix} a_{4,1} \\ a_{4,2} \\ a_{4,3} \\ \vdots \\ a_{4,16} \end{pmatrix}. \tag{32}$$

Here, the degenerated ground state $\left|\phi_i\right\rangle(i = 1,2,3,4)$ is obtained from the diagonalization of system Hamiltonian directly. We find that some coefficients in the ground state $\left|\Xi\right\rangle_4$ are same (details are addressed in Supplementary Table 7), other coefficients $a_{4,i}$ ($a_{4,2}, a_{4,3}, a_{4,5}, a_{4,8}, a_{4,9}, a_{4,12}, a_{4,14}, a_{4,15}$) take zero.

When there are 8 spins in the square lattice, we provide the detailed forms of system with 8 spins as

$$\begin{aligned} H_{\mathrm{t},8} = &-(\sigma_x^1\sigma_x^2\sigma_x^5\sigma_x^6 + \sigma_x^2\sigma_x^3\sigma_x^6\sigma_x^7 + \sigma_x^3\sigma_x^7\sigma_x^4\sigma_x^8 + \sigma_x^4\sigma_x^8\sigma_x^1\sigma_x^5 \\ &+\sigma_z^1\sigma_z^2\sigma_z^5\sigma_z^6 + \sigma_z^2\sigma_z^3\sigma_z^6\sigma_z^7 + \sigma_z^3\sigma_z^4\sigma_z^7\sigma_z^8 + \sigma_z^4\sigma_z^8\sigma_z^1\sigma_z^5) \\ &-g\left(\sigma_x^1 + \sigma_x^2 + \sigma_x^3 + \sigma_x^4 + \sigma_x^5 + \sigma_x^6 + \sigma_x^7 + \sigma_x^8 + \sigma_z^1 + \sigma_z^2 + \sigma_z^3 + \sigma_z^4 + \sigma_z^5 + \sigma_z^6 + \sigma_z^7 + \sigma_z^8\right). \end{aligned} \tag{33}$$

The schematic representation is shown in Supplementary Figure 12. We express the states with 8 spins as

$$\left|\Phi\right\rangle_8 = a_{8,1}\left|00000000\right\rangle + a_{8,2}\left|00000001\right\rangle + a_{8,3}\left|00000010\right\rangle + \cdots a_{8,256}\left|11111111\right\rangle, \tag{34}$$

which can be written as

$$\left|\Phi\right\rangle_8 = \begin{pmatrix} a_{8,1} \\ a_{8,2} \\ a_{8,3} \\ \vdots \\ a_{8,256} \end{pmatrix}. \tag{35}$$

When the system belongs to topologically trivial phase ($g > 0.34$), there are no nearly degenerated ground states and the topological properties can be revealed from its unique ground state. In order to describe the topological properties of system with external fields, we need to obtain the corresponding ground states for systems. Since there are no analytic forms of ground states for the system with external fields, we numerically obtain the ground states of system ($\left|\Phi_g\right\rangle_8$) and list as below. When the system has 8 spins and $g \geq 0.9$, we find that some coefficients in the ground state $\left|\Phi_g\right\rangle_8$ are same, that is

(1) $a_{8,1}$,

(2) $a_{8,2} = a_{8,3} = a_{8,5} = a_{8,9} = a_{8,17} = a_{8,33} = a_{8,65} = a_{8,129}$,

$$(3)\quad \begin{aligned}&a_{8,4}=a_{8,7}=a_{8,10}=a_{8,13}=a_{8,19}=a_{8,25}=a_{8,34}=a_{8,37}\\&=a_{8,49}=a_{8,67}=a_{8,73}=a_{8,97}=a_{8,130}=a_{8,133}=a_{8,145}=a_{8,193}\end{aligned},$$

$$(4)\quad a_{8,6}=a_{8,11}=a_{8,21}=a_{8,41}=a_{8,66}=a_{8,81}=a_{8,131}=a_{8,161},$$

$$(5)\quad \begin{aligned}&a_{8,8}=a_{8,12}=a_{8,14}=a_{8,15}=a_{8,23}=a_{8,27}=a_{8,29}=a_{8,38}=a_{8,42}=a_{8,45}=a_{8,53}=a_{8,57}\\&=a_{8,68}=a_{8,74}=a_{8,75}=a_{8,83}=a_{8,89}=a_{8,98}=a_{8,105}=a_{8,113}=a_{8,132}=a_{8,134}=a_{8,135}\\&=a_{8,147}=a_{8,149}=a_{8,162}=a_{8,165}=a_{8,177}=a_{8,194}=a_{8,195}=a_{8,209}=a_{8,225}\end{aligned},$$

$$(6)\quad \begin{aligned}&a_{8,16}=a_{8,31}=a_{8,46}=a_{8,61}=a_{8,76}=a_{8,91}=a_{8,106}=a_{8,121}\\&=a_{8,136}=a_{8,151}=a_{8,166}=a_{8,181}=a_{8,196}=a_{8,211}=a_{8,226}=a_{8,241}\end{aligned},$$

$$(7)\quad a_{8,18}=a_{8,35}=a_{8,69}=a_{8,137},$$

$$(8)\quad \begin{aligned}&a_{8,20}=a_{8,26}=a_{8,36}=a_{8,39}=a_{8,50}=a_{8,51}=a_{8,71}=a_{8,77}\\&=a_{8,99}=a_{8,101}=a_{8,138}=a_{8,141}=a_{8,146}=a_{8,153}=a_{8,197}=a_{8,201}\end{aligned},$$

$$(9)\quad a_{8,22}=a_{8,43}=a_{8,70}=a_{8,82}=a_{8,85}=a_{8,139}=a_{8,163}=a_{8,169},$$

$$(10)\quad \begin{aligned}&a_{8,24}=a_{8,30}=a_{8,44}=a_{8,47}=a_{8,54}=a_{8,59}=a_{8,72}=a_{8,78}=a_{8,84}=a_{8,87}=a_{8,90}=a_{8,93}\\&=a_{8,102}=a_{8,107}=a_{8,114}=a_{8,117}=a_{8,140}=a_{8,143}=a_{8,150}=a_{8,155}=a_{8,164}=a_{8,167}=a_{8,170}\\&=a_{8,173}=a_{8,179}=a_{8,185}=a_{8,198}=a_{8,203}=a_{8,210}=a_{8,213}=a_{8,227}=a_{8,233}\end{aligned},$$

$$(11)\quad \begin{aligned}&a_{8,28}=a_{8,40}=a_{8,55}=a_{8,58}=a_{8,79}=a_{8,100}=a_{8,109}=a_{8,115}\\&=a_{8,142}=a_{8,148}=a_{8,157}=a_{8,178}=a_{8,199}=a_{8,202}=a_{8,217}=a_{8,229}\end{aligned},$$

$$(12)\quad \begin{aligned}&a_{8,32}=a_{8,48}=a_{8,62}=a_{8,63}=a_{8,80}=a_{8,92}=a_{8,95}=a_{8,108}=a_{8,110}=a_{8,122}=a_{8,123}=a_{8,125}\\&=a_{8,144}=a_{8,152}=a_{8,159}=a_{8,168}=a_{8,174}=a_{8,182}=a_{8,183}=a_{8,189}=a_{8,200}=a_{8,204}=a_{8,212}\\&=a_{8,215}=a_{8,219}=a_{8,228}=a_{8,230}=a_{8,234}=a_{8,242}=a_{8,243}=a_{8,245}=a_{8,249}\end{aligned},$$

$$(13)\quad a_{8,52}=a_{8,103}=a_{8,154}=a_{8,205},$$

$$(14)\quad \begin{aligned}&a_{8,56}=a_{8,60}=a_{8,104}=a_{8,111}=a_{8,116}=a_{8,119}=a_{8,156}=a_{8,158}\\&=a_{8,180}=a_{8,186}=a_{8,206}=a_{8,207}=a_{8,218}=a_{8,221}=a_{8,231}=a_{8,237}\end{aligned},$$

$$(15)\quad \begin{aligned}&a_{8,64}=a_{8,112}=a_{8,124}=a_{8,127}=a_{8,160}=a_{8,184}=a_{8,190}=a_{8,208}\\&=a_{8,220}=a_{8,223}=a_{8,232}=a_{8,238}=a_{8,244}=a_{8,247}=a_{8,250}=a_{8,253}\end{aligned},$$

$$(16)\quad a_{8,86}=a_{8,171},$$

$$(17)\quad a_{8,88}=a_{8,94}=a_{8,118}=a_{8,172}=a_{8,175}=a_{8,187}=a_{8,214}=a_{8,235},$$

$$(18)\quad a_{8,96}=a_{8,126}=a_{8,176}=a_{8,191}=a_{8,216}=a_{8,236}=a_{8,246}=a_{8,251},$$

$$(19)\quad a_{8,120}=a_{8,188}=a_{8,222}=a_{8,239},$$

$$(20)\quad a_{8,128}=a_{8,192}=a_{8,224}=a_{8,240}=a_{8,248}=a_{8,252}=a_{8,254}=a_{8,255},$$

$$(21)\quad a_{8,256}. \tag{36}$$

The coefficients $a_{8,i}(i=1,\cdots,256)$ for the ground states of system $\left|\Phi_g\right\rangle_8$ with different strengths of external fields are listed in Supplementary Table 8.

When the system has 8 spins and $g \le 0.34$, the system belongs to the $Z_2$ phase and there exist four nearly degenerated ground states. By using the numerical diagonalization to the system Hamiltonian, we can obtain such

four nearly degenerated ground states. We do the linear combination for these four nearly degenerated ground states and obtain the MES. Here we present the expression for the MES with $g=0.1$ as

$$\left|\Xi\right\rangle_8 = 0.5\left|\phi_1\right\rangle + \frac{1}{\sqrt{2}}\left|\phi_2\right\rangle + 0.5\left|\phi_4\right\rangle = \begin{pmatrix} a_{8,1} \\ a_{8,2} \\ a_{8,3} \\ \vdots \\ a_{8,256} \end{pmatrix}. \tag{37}$$

Here, the nearly degenerated ground state $\left|\phi_i\right\rangle(i=1,2,3,4)$ is obtained from the diagonalization to system Hamiltonian directly. The coefficients $a_{8,i}(i=1,\dots,256)$ for the MES $\left|\Xi\right\rangle_8$ are

(1) $a_{8,2}=a_{8,3}=a_{8,5}=a_{8,9}=a_{8,17}=a_{8,33}=a_{8,65}=a_{8,129}=-0.0071$,

(2) $a_{8,4}=a_{8,7}=a_{8,10}=a_{8,13}=a_{8,19}=a_{8,25}=a_{8,34}=a_{8,37}=a_{8,49}=a_{8,67}=a_{8,73}=a_{8,97}$ $=a_{8,130}=a_{8,133}=a_{8,145}=a_{8,193}=-0.0014$,

(3) $a_{8,8}=a_{8,12}=a_{8,14}=a_{8,15}=a_{8,23}=a_{8,27}=a_{8,29}=a_{8,38}=a_{8,42}=a_{8,45}=a_{8,53}=a_{8,57}=a_{8,68}$ $=a_{8,74}=a_{8,75}=a_{8,83}=a_{8,89}=a_{8,98}=a_{8,105}=a_{8,113}=a_{8,132}=a_{8,134}=a_{8,135}=a_{8,147}=a_{8,149}$ $=a_{8,162}=a_{8,165}=a_{8,177}=a_{8,194}=a_{8,195}=a_{8,209}=a_{8,225}=-0.0066$,

(4) $a_{8,18}=a_{8,35}=a_{8,69}=a_{8,137}=-0.2710$,

(5) $a_{8,20}=a_{8,22}=a_{8,26}=a_{8,36}=a_{8,39}=a_{8,43}=a_{8,50}=a_{8,51}=a_{8,70}=a_{8,71}=a_{8,77}=a_{8,82}$ $=a_{8,85}=a_{8,99}=a_{8,101}=a_{8,138}=a_{8,139}=a_{8,141}=a_{8,146}=a_{8,153}=a_{8,163}=a_{8,169}=a_{8,197}$ $=a_{8,201}=-0.0067$,

(6) $a_{8,24}=a_{8,30}=a_{8,44}=a_{8,47}=a_{8,54}=a_{8,59}=a_{8,64}=a_{8,72}=a_{8,78}=a_{8,84}=a_{8,87}=a_{8,90}$ $=a_{8,93}=a_{8,102}=a_{8,107}=a_{8,112}=a_{8,114}=a_{8,117}=a_{8,124}=a_{8,127}=a_{8,140}=a_{8,143}=a_{8,150}$ $=a_{8,155}=a_{8,160}=a_{8,164}=a_{8,167}=a_{8,170}=a_{8,173}=a_{8,179}=a_{8,184}=a_{8,185}=a_{8,190}=a_{8,198}$ $=a_{8,203}=a_{8,208}=a_{8,210}=a_{8,213}=a_{8,220}=a_{8,223}=a_{8,227}=a_{8,232}=a_{8,233}=a_{8,238}=a_{8,244}$ $=a_{8,247}=a_{8,250}=a_{8,253}=-0.0012$,

(7) $a_{8,31}=a_{8,46}=a_{8,76}=a_{8,121}=a_{8,136}=a_{8,181}=a_{8,211}=a_{8,226}=-0.2509$,

(8) $a_{8,32}=a_{8,48}=a_{8,62}=a_{8,63}=a_{8,80}=a_{8,92}=a_{8,95}=a_{8,108}=a_{8,110}=a_{8,122}=a_{8,123}=a_{8,125}$ $=a_{8,144}=a_{8,152}=a_{8,159}=a_{8,168}=a_{8,174}=a_{8,182}=a_{8,183}=a_{8,189}=a_{8,200}=a_{8,204}=a_{8,212}$ $=a_{8,215}=a_{8,219}=a_{8,228}=a_{8,230}=a_{8,234}=a_{8,242}=a_{8,243}=a_{8,245}=a_{8,249}=-0.0062$

(9) $a_{8,56}=a_{8,60}=a_{8,88}=a_{8,94}=a_{8,104}=a_{8,111}=a_{8,116}=a_{8,118}=a_{8,119}=a_{8,156}=a_{8,158}$ $=a_{8,172}=a_{8,175}=a_{8,180}=a_{8,186}=a_{8,187}=a_{8,206}=a_{8,207}=a_{8,214}=a_{8,218}=a_{8,221}$ $=a_{8,231}=a_{8,235}=a_{8,237}=-0.0058$,

(10) $a_{8,120}=a_{8,188}=a_{8,222}=a_{8,239}=-0.2222$,

(11) $a_{8,128}=a_{8,192}=a_{8,224}=a_{8,240}=a_{8,248}=a_{8,252}=a_{8,254}=a_{8,255}=-0.0055$. (38)

Other coefficients $a_{8,i}$ can take zero. When the strength takes $g=0.2$, we can obtain the MES by doing linear combination of four nearly degenerated ground states as

$$|\Xi\rangle_8 = 0.6|\phi_1\rangle + 0.65|\phi_3\rangle + 0.4664|\phi_4\rangle = \begin{pmatrix} a_{8,1} \\ a_{8,2} \\ a_{8,3} \\ \vdots \\ a_{8,256} \end{pmatrix}. \quad (39)$$

Here, the nearly degenerated ground state $|\phi_i\rangle(i=1,2,3,4)$ is obtained from the diagonalization of system Hamiltonian directly. Some coefficients $a_{8,i}(i=1,...,256)$ in the MES $|\Xi\rangle_8$ are same, which are addressed below

(1) $a_{8,1} = -0.3829$,

(2) $a_{8,2} = a_{8,3} = a_{8,5} = a_{8,9} = a_{8,17} = a_{8,33} = a_{8,65} = a_{8,129} = -0.0197$,

(3) $a_{8,4} = a_{8,7} = a_{8,10} = a_{8,13} = a_{8,19} = a_{8,25} = a_{8,34} = a_{8,37} = a_{8,49} = a_{8,67} = a_{8,73} = a_{8,97} = a_{8,130} = a_{8,133} = a_{8,145} = a_{8,193} = -0.0060$,

(4) $a_{8,6} = a_{8,11} = a_{8,21} = a_{8,41} = a_{8,66} = a_{8,81} = a_{8,131} = a_{8,161} = -0.0030$,

(5) $a_{8,8} = a_{8,12} = a_{8,14} = a_{8,15} = a_{8,23} = a_{8,27} = a_{8,29} = a_{8,38} = a_{8,42} = a_{8,45} = a_{8,53} = a_{8,57} = a_{8,68} = a_{8,74} = a_{8,75} = a_{8,83} = a_{8,89} = a_{8,98} = a_{8,105} = a_{8,113} = a_{8,132} = a_{8,134} = a_{8,135} = a_{8,147} = a_{8,149} = a_{8,162} = a_{8,165} = a_{8,177} = a_{8,194} = a_{8,195} = a_{8,209} = a_{8,225} = -0.0133$,

(6) $a_{8,16} = a_{8,61} = a_{8,91} = a_{8,106} = a_{8,151} = a_{8,166} = a_{8,196} = a_{8,241} = -0.2354$,

(7) $a_{8,18} = a_{8,35} = a_{8,69} = a_{8,137} = -0.0164$,

(8) $a_{8,20} = a_{8,26} = a_{8,36} = a_{8,39} = a_{8,50} = a_{8,51} = a_{8,71} = a_{8,77} = a_{8,99} = a_{8,101} = a_{8,138} = a_{8,141} = a_{8,146} = a_{8,153} = a_{8,197} = a_{8,201} = -0.0144$,

(9) $a_{8,22} = a_{8,43} = a_{8,70} = a_{8,82} = a_{8,85} = a_{8,139} = a_{8,163} = a_{8,169} = -0.0139$,

(10) $a_{8,24} = a_{8,30} = a_{8,44} = a_{8,47} = a_{8,54} = a_{8,59} = a_{8,72} = a_{8,78} = a_{8,84} = a_{8,87} = a_{8,90} = a_{8,93} = a_{8,102} = a_{8,107} = a_{8,114} = a_{8,117} = a_{8,140} = a_{8,143} = a_{8,150} = a_{8,155} = a_{8,164} = a_{8,167} = a_{8,170} = a_{8,173} = a_{8,179} = a_{8,185} = a_{8,198} = a_{8,203} = a_{8,210} = a_{8,213} = a_{8,227} = a_{8,233} = -0.0050$,

(11) $a_{8,28} = a_{8,40} = a_{8,55} = a_{8,58} = a_{8,79} = a_{8,100} = a_{8,109} = a_{8,115} = a_{8,142} = a_{8,148} = a_{8,157} = a_{8,178} = a_{8,199} = a_{8,202} = a_{8,217} = a_{8,229} = -0.0026$,

(12) $a_{8,31} = a_{8,46} = a_{8,76} = a_{8,96} = a_{8,121} = a_{8,126} = a_{8,136} = a_{8,176} = a_{8,181} = a_{8,191} = a_{8,211} = a_{8,216} = a_{8,226} = a_{8,236} = a_{8,246} = a_{8,251} = -0.0022$,

(13) $a_{8,32} = a_{8,48} = a_{8,62} = a_{8,63} = a_{8,80} = a_{8,92} = a_{8,95} = a_{8,108} = a_{8,110} = a_{8,122} = a_{8,123} = a_{8,125} = a_{8,144} = a_{8,152} = a_{8,159} = a_{8,168} = a_{8,174} = a_{8,182} = a_{8,183} = a_{8,189} = a_{8,200} = a_{8,204} = a_{8,212} = a_{8,215} = a_{8,219} = a_{8,228} = a_{8,230} = a_{8,234} = a_{8,242} = a_{8,243} = a_{8,245} = a_{8,249} = -0.0120$,

(14) $a_{8,52} = a_{8,103} = a_{8,154} = a_{8,205} = -0.2464$,

(15) $a_{8,56} = a_{8,60} = a_{8,104} = a_{8,111} = a_{8,116} = a_{8,119} = a_{8,156} = a_{8,158} = a_{8,180} = a_{8,186} = a_{8,206} = a_{8,207} = a_{8,218} = a_{8,221} = a_{8,231} = a_{8,237} = -0.0129$,

(16) $a_{8,64} = a_{8,112} = a_{8,124} = a_{8,127} = a_{8,160} = a_{8,184} = a_{8,190} = a_{8,208} = a_{8,220} = a_{8,223} = a_{8,232} = a_{8,238} = a_{8,244} = a_{8,247} = a_{8,250} = a_{8,253} = -0.0043$,

(17) $a_{8,86} = a_{8,171} = -0.2365$,

(18) $a_{8,88} = a_{8,94} = a_{8,118} = a_{8,172} = a_{8,175} = a_{8,187} = a_{8,214} = a_{8,235} = -0.0125$,

(19) $a_{8,120} = a_{8,188} = a_{8,222} = a_{8,239} = -0.0116$,

(20) $a_{8,128} = a_{8,192} = a_{8,224} = a_{8,240} = a_{8,248} = a_{8,252} = a_{8,254} = a_{8,255} = -0.0100$,

(21) $a_{8,256} = -0.1710$. (40)

When we have obtained the entanglement entropies for MESs with different strengths $g$, we can extract the TEE from these entropies and show in Fig. 5 of main text.

## Supplementary Note 7. The construction of MESs for toric code model with symmetrically applied fields

Here, we add the external fields along x and z directions to the pure toric code model. The system Hamiltonian changes to

$$H^{\mathrm{tc}}{}_{\mathrm{t}} = H^{\mathrm{tc}}{}_{0} + H^{\mathrm{tc}}{}_{1}, \tag{41}$$

where $H_0$ describes the pure toric code model without external fields. The Hamiltonian $H_1$ represents the external fields and takes the form as

$$H^{\mathrm{tc}}{}_{1} = -g\sum_{i=1}^{n}(\sigma_x^i + \sigma_z^i). \tag{42}$$

The strength $g$ represents the strength of symmetrically external fields. The subscript *i* is chosen for all the spins on the lattice. In this case, the Hamiltonian for such model with 8 spins is

$$\begin{aligned} H^{\mathrm{tc}}{}_{\mathrm{t},8} &= -\sigma_x^1\sigma_x^3\sigma_x^2\sigma_x^7 - \sigma_z^1\sigma_z^3\sigma_z^4\sigma_z^5 - \sigma_x^5\sigma_x^3\sigma_x^7\sigma_x^6 - \sigma_z^5\sigma_z^8\sigma_z^7\sigma_z^1 \\ &-\sigma_z^2\sigma_z^3\sigma_z^4\sigma_z^6 - \sigma_x^1\sigma_x^2\sigma_x^4\sigma_x^8 - \sigma_z^6\sigma_z^7\sigma_z^8\sigma_z^2 - \sigma_x^4\sigma_x^5\sigma_x^6\sigma_x^8 \\ &-g\left(\sigma_x^1 + \sigma_x^2 + \sigma_x^3 + \sigma_x^4 + \sigma_x^5 + \sigma_x^6 + \sigma_x^7 + \sigma_x^8 + \sigma_z^1 + \sigma_z^2 + \sigma_z^3 + \sigma_z^4 + \sigma_z^5 + \sigma_z^6 + \sigma_z^7 + \sigma_z^8\right). \end{aligned} \tag{43}$$

Similar as the discussion without external fields above, we separate this system into two subsystems (details in Supplementary Figure 13(a)), one contains the spin 1, 2, 3 and 4, and the other contains the spin 5, 6, 7 and 8. When considering the study with external fields, we choose different strengths *g* which changes from 0.1 to 10.

When adding the external fields to the system with 12 spins, we can obtain the Hamiltonian for the toric code model as

$$\begin{aligned} H^{\mathrm{tc}}{}_{\mathrm{t},12} &= -\sigma_x^1\sigma_x^{10}\sigma_x^4\sigma_x^3 - \sigma_z^1\sigma_z^{10}\sigma_z^{11}\sigma_z^7 - \sigma_x^4\sigma_x^7\sigma_x^{10}\sigma_x^9 - \sigma_z^1\sigma_z^4\sigma_z^5\sigma_z^7 - \sigma_x^1\sigma_x^2\sigma_x^5\sigma_x^{11} - \sigma_z^2\sigma_z^8\sigma_z^{11}\sigma_z^{12} \\ &-\sigma_z^2\sigma_z^5\sigma_z^6\sigma_z^8 - \sigma_x^5\sigma_x^7\sigma_x^8\sigma_x^{11} - \sigma_z^3\sigma_z^4\sigma_z^6\sigma_z^9 - \sigma_x^2\sigma_x^3\sigma_x^6\sigma_x^{12} - \sigma_z^9\sigma_z^3\sigma_z^{10}\sigma_z^{12} - \sigma_x^6\sigma_x^8\sigma_x^9\sigma_x^{12} \\ &-g\left(\sigma_x^1 + \sigma_x^2 + \sigma_x^3 + \sigma_x^4 + \sigma_x^5 + \sigma_x^6 + \sigma_x^7 + \sigma_x^8 + \sigma_x^9 + \sigma_x^{10} + \sigma_x^{11} + \sigma_x^{12}\right) \\ &-g\left(\sigma_z^1 + \sigma_z^2 + \sigma_z^3 + \sigma_z^4 + \sigma_z^5 + \sigma_z^6 + \sigma_z^7 + \sigma_z^8 + \sigma_z^9 + \sigma_z^{10} + \sigma_z^{11} + \sigma_z^{12}\right). \end{aligned} \tag{44}$$

Similar as the discussion without external fields above, we separate this system into two subsystems (details in Supplementary Figure 13(b)), one contains the spin 1, 2, 3, 4, 5 and 6, and the other contains the spin 7, 8, 9, 10, 11 and 12. When considering the study with external fields, we choose different strengths *g* which changes from 0.1 to 10.

When the external fields along x and z directions are added to the system, there are no analytic expressions of

ground states for this toric code model. When $g < 0.34$, the system belongs to the $Z_2$ topological phase and there exists four nearly degenerated ground states in the system; when $g > 0.34$, the system belongs to the topologically trivial phase and there is only one ground state in the system. The MES for the toric code model in the $Z_2$ topological phase can be obtained by the linear combination of four nearly degenerated ground states; while the MES for the system in the topologically trivial phase is just the ground state itself. The entanglement entropies for the MES of system containing 8 or 12 spins are listed in Supplementary Table 9.

With these entanglement entropies for 8 and 12-spins toric code models with different strengths $g$, we can extract the TEE value for this model in the figure below. As shown in Supplementary Figure 14, the transition from $Z_2$ topological phase to trivial phase in the toric code model agrees well with that in the square lattice.

## Supplementary Note 8. The detailed descriptions on the experiments for 4- and 8-cebit analogies of states with external fields

In Fig. 3(b) of main text and Supplementary Note 4, we have presented the processes for demonstrating the 4-cebit CMES $|\psi_4^{\mathrm{cl}}\rangle$ and 8-cebit $|\psi_8^{\mathrm{cl}}\rangle$, respectively. By contrast, the ground states of the Hamiltonian with external fields in Supplementary Equation 25 possess more complicated structures. Therefore, we need to design new specific circuits according to the concrete form of each state.

**Experimental details for constructing 4-cebit analogies of states with a small $g$.** In Supplementary Figure 15 (i.e. Fig. 4 of main text), we present the scheme for the analogy of $|\Xi\rangle_4$ in Supplementary Equation 31 with a small $g$=0.1. The down-converted frequencies of the 8 signals in channels $\{h_1, v_1, \cdots h_4, v_4\}$ through DDC are set as

$$\begin{aligned} &f_{h_1} = 1.3\text{MHz}, f_{v_1} = 1.8\text{MHz}, f_{h_2} = 2.7\text{MHz}, f_{v_2} = 2.3\text{MHz}, \\ &f_{h_3} = 3.7\text{MHz}, f_{v_3} = 3.3\text{MHz}, f_{h_4} = 4.2\text{MHz}, f_{v_4} = 4.7\text{MHz}. \end{aligned} \tag{45}$$

and then $\{B_{h_1}, B_{v_1}, \cdots, B_{h_4}, B_{v_4}\}$ go through the PROJ followed by a series of mixing and filtering processes.

First, the recombined signals $S_1$ and $S_2$ are mixed by the multiplier $M_1$ and then two bandpass filters $F_1$ (passband: 3.9—4.2MHz) and $F_2$ (passband: 0.8—1.1MHz), respectively. The former selects two sum-frequency components at 4MHz indicating the $|h_1h_2\rangle$ term and 4.1MHz indicating the $|v_1v_2\rangle$ term, while the latter selects two difference-frequency components at 1.0MHz indicating the $|h_1v_2\rangle$ term and 0.9MHz indicating the $|v_1h_2\rangle$ term. These four frequency components are recombined into a mixed signal $S_{12}$ by an adder. Similarly, the signals $S_3$ and $S_4$ go through a multiplier $M_2$, two filters $F_3$ (passband: 7.8—8.1MHz) and $F_4$ (passband: 0.8—1.1MHz) so that four distinct frequency components at 7.9MHz ($|h_3h_4\rangle$), 8.0MHz ($|v_3v_4\rangle$), 1.0MHz ($|h_3v_4\rangle$), and 0.9MHz ($|v_3h_4\rangle$) are selected and then recombined into the mixed signal $S_{34}$.

Then, the two summed product signals $S_{12}$ and $S_{34}$ are mixed by a multiplier $M_3$, followed by a collection of FFT-based digital filters $\{FFT_1, \ldots, FFT_6\}$ to select 6 desired frequency components corresponding to those superposed terms included in Supplementary Table 7. The associated modulators $\{d_1, \ldots, d_5\}$ are used for adjusting the complex amplitudes of these filtered components according to the expanded coefficients in Supplementary Table 7. The parameters for the FFT filters and modulators with their corresponding terms are listed in Supplementary Table 10.

The advantage of our designed process is that only the modulators at the bottom of Supplementary Figure 15

need to be adjusted for the analogy of other states with different small values of $g$. For example, we set $d_1$=0, $d_2$= -0.0104, $d_3$=0.5051, $d_4$= -0.4947, and $d_5$=0 for the case of $g$=0.2 in Supplementary Equation 32. After recording the desired joint complex amplitude denoted as *A*, by exploiting the tomography methods for characterizing these analogies of 4-qubit states as introduced in Supplementary Note 3, we obtain the average fidelities of these 4-cebit analog states with $g = 0.1,\ \ 0.2$ as $0.9973 \pm 0.0010$, $0.9978 \pm 0.0012$ respectively.

**Experimental details for constructing 4-cebit analogies of states with a large $g$.** Now, we turn to the analogies of states with a large g by a simple modification of Supplementary Figure 15. For simulating the state $\left|\Phi_{g}\right\rangle_{4}$ with a large $g = 10$, we keep the settings in PROJ and multipliers $M_1$ and $M_2$ unchanged as shown in Supplementary Figure 16. Next, the filters $F_1$ and $F_2$ in Supplementary Figure 15 are replaced by a filter $F_{12}$ with a passband as 3.0—5.0MHz to select four sum frequency terms: $|h_1 h_2)$ at 4.0MHz, $|h_1 v_2)$ at 3.6MHz, $|v_1 h_2)$ at 4.5MHz, and $|v_1 v_2)$ at 4.1MHz, which are then recombined into a mixed signal $S_{12}$. Similarly, the original $F_3$ and $F_4$ are replaced by $F_{34}$ with a passband as 7.0—9.0MHz to select four sum frequency terms: $|h_3 h_4)$ at 7.9MHz, $|h_3 v_4)$ at 8.4MHz, $|v_3 h_4)$ at 7.5MHz, and $|v_3 v_4)$ at 8.0MHz, which are then recombined into a mixed signal $S_{34}$.

Then, the two summed product signals $S_{12}$ and $S_{34}$ are mixed by a multiplier $M_3$, followed by a collection of FFT-based digital filters $\{FFT_1,\ldots,FFT_9\}$ to select 9 desired frequency components corresponding to those superposed terms in Supplementary Equation 28. The associated modulators $\{d_1,\ldots,d_5\}$ are used for adjusting the complex amplitudes of these filtered components according to the expanded coefficients in Supplementary Table 6. The parameters for the FFT filters and modulators with their corresponding terms are listed in Supplementary Table 11.

For the analogies of other states with different large values of $g = 0.9,1,2,5,10$, only the modulators at the bottom of Supplementary Figure 16 need to be adjusted according to Supplementary Table 6. After recording the desired joint complex amplitude denoted as *A*, by exploiting the tomography methods for characterizing these analogies of 4-qubit states as introduced in Supplementary Note 3, we obtain the average fidelities of these 4-cebit analog states with $g = 0.9,1,2,5,10$ as $0.9962 \pm 0.0020$, $0.9955 \pm 0.0014$, $0.9959 \pm 0.0013$, $0.9952 \pm 0.0011$, $0.9975 \pm 0.0006$ respectively.

**Experimental details for constructing 8-cebit analogies of states with applied fields.** In this part, we consider how to demonstrate the analogy of the 8-qubit states with external fields as $\left|\Phi_{g}\right\rangle_{8}$ in Supplementary Equation 34 and $\left|\Xi\right\rangle_{8}$ in Supplementary Equations 37 and 39.

The circuit designed in the DSP module for the cases with a large g (=10,5,2,1,0.9) is shown in Supplementary Figure 17. The incoming signals $\{B_{h_1}, B_{v_1}, \cdots, B_{h_8}, B_{v_8}\}$, the PROJ part and the first filtering stage are the same as those in Supplementary Figure 9, while the settings in the second stage are modified. The filters $F_5$ (passband: 6.8—18.0MHz) and $F_6$ (passband: 10.0—24.0 MHz) are used to select 16 distinct frequency components indicating $\{\,|h_1 h_2 h_3 h_4),\ldots,|v_1 v_2 v_3 v_4)\,\}$ and $\{\,|h_5 h_6 h_7 h_8),\ldots,|v_5 v_6 v_7 v_8)\,\}$ as listed in Supplementary Table 12, respectively.

In the following, the two summed signals $S_{1-4}$ and $S_{5-8}$ composed of these frequency components $\{|h_1h_2h_3h_4), \ldots, |v_1v_2v_3v_4)\}$ and $\{|h_5h_6h_7h_8), \ldots, |v_5v_6v_7v_8)\}$ listed in Supplementary Table 12 are mixed by a multiplier $M_7$ as shown in Supplementary Figure 17. Then the combined signal passes through a collection of FFT-based digital filters $\{FFT_1, \ldots, FFT_{256}\}$ where 256 amplitudes $\{A_{\omega_1}, A_{\omega_2} \ldots, A_{\omega_{256}}\}$ of the desired frequency components are selected respectively corresponding to those 256 superposed terms in Supplementary Equation 34. The associated modulators $\{d_1, \ldots, d_{21}\}$ are used for adjusting the complex amplitudes of these filtered components according to the expanded coefficients in Supplementary Table 8. The filtered frequencies, the corresponding terms and the amplitude modulation are listed in Supplementary Table 13.

For the analogies of other states with different small values of $g = 0.9, 1, 2, 5, 10$, only the modulators at the bottom of Supplementary Figure 17 need to be adjusted according to Supplementary Table 8. Also, the design for the cases with a small g (=0.1,0.2) are similar to Supplementary Figure 17, where only the FFT-based filters and modulators need to be adjusted according to the coefficients for the state $|\Xi\rangle_8$. For example, to observe an analogy of $|\Xi\rangle_8$ with g=0.2 as in Supplementary Equation 39, we can simply rearrange all 256 filtered frequency components and their associated modulators listed in Supplementary Table 13 according to the expanded coefficients in Supplementary Equation 40, and then recorded final results. Similarly, the analogy for the case with g=0.1 in Supplementary Equation 37 can also be obtained with slight modifications of such a circuit. Then, by exploiting the tomography methods for characterizing these analogies of 8-qubit states as introduced in Supplementary Note 5, we obtain the average fidelities of these 8-cebit analog states with $g = 0.1, 0.2, 0.9, 1, 2, 5, 10$ as $0.9856 \pm 0.0033$, $0.9815 \pm 0.0065$, $0.9692 \pm 0.0040$, $0.9724 \pm 0.0025$, $0.9699 \pm 0.0044$, $0.9800 \pm 0.0054$, $0.9864 \pm 0.0040$ respectively.

## Supplementary References


1. Y. Zhang, T. Grover, A. Turner, M. Oshikawa, and A. Vishwanath, Physical Review B 85, 235151 (2012).
2. S. C. Morampudi, C. von Keyserlingk, and F. Pollmann, Physical Review B 90, 035117 (2014).
3. Jones, R. C. Journal of the Optical Society of America 31, 488 (1941).
4. R. J. C. Spreeuw, Physical Review A 63, 062302 (2001).
5. R. J. C. Spreeuw, Foundations of physics 28, 361 (1998).
6. P. Walther, K. J. Resch, T. Rudolph, E. Schenck, H. Weinfurter, V. Vedral, M. Aspelmeyer, and A. Zeilinger, Nature 434, 169 (2005).
7. K. J. Resch, P. Walther, and A. Zeilinger, Physical Review Letters 94, 070402 (2005).
8. N. Kiesel, C. Schmid, U. Weber, G. Toth, O. Guhne, R. Ursin, and H. Weinfurter, Physical Review Letters 95, 210502 (2005).
9. M. D. de Burgh, N. K. Langford, A. C. Doherty, and A. Gilchrist, Physical Review A 78, 052122 (2008).
10. B. Qi, Z. Hou, L. Li, D. Dong, G. Xiang, and G. Guo, Scientific Reports 3, 3496 (2013).
11. J. A. Smolin, J. M. Gambetta, and G. Smith, Physical Review Letters 108, 070502 (2012).
12. D. L. Donoho, IEEE Transactions on information theory **52**, 1289 (2006).
13. E. J. Candès, J. Romberg, and T. Tao, IEEE Transactions on information theory 52, 489 (2006).

14. E. J. Candes and T. Tao, IEEE transactions on information theory 52, 5406 (2006).
15. D. Gross, Y.-K. Liu, S. T. Flammia, S. Becker, and J. Eisert, Physical Review Letters 105, 150401 (2010).
16. S. T. Flammia, D. Gross, Y. Liu, and J. Eisert, New Journal of Physics 14, 095022 (2012).
17. A. Steffens, C. Riofrío, W. McCutcheon, I. Roth, B. Bell, A. McMillan, M. Tame, J. Rarity, and J. Eisert, Quantum Science and Technology 2, 025005 (2017).
18. M. Grant, S. Boyd, CVX: Matlab software for disciplined convex programming, version 2.0 beta. http://cvxr.com/cvx, September 2013.
